# Function and regulation of *pob* genes for 4-hydroxybenzoate catabolism in *Agrobacterium tumefaciens*

**DOI:** 10.1128/aem.00255-25

**Published:** 2025-06-03

**Authors:** Nan Xu, Wanyu Wang, Shuang Cheng, Jiaojiao Zuo, Minliang Guo

**Affiliations:** 1 College of Bioscience and Biotechnology, Yangzhou University 614690, Yangzhou, China; The University of Tennessee Knoxville, Knoxville, Tennessee, USA

**Keywords:** *Agrobacterium tumefaciens*, 4-hydroxybenzoate metabolism, β-ketoadipate pathway, *pobR*, *pobA*

## Abstract

*Agrobacterium tumefaciens* is a pathogen that causes tumors in plants. Phenolic acids present in the soil and rhizosphere may affect the interaction between *A. tumefaciens* and plants. An important pathway for microorganisms to degrade phenolic acids is the β-ketoadipate pathway, which has been annotated in the genome of *A. tumefaciens*. The ability of the PobA (atu4544) enzyme to catalyze the conversion of 4-hydroxybenzoate to protocatechuate was essential for cell growth using 4-hydroxybenzoate as the sole carbon source. The *pobA* gene is located upstream of *atu4545*, encoding an AraC transcription factor (PobR). Strains with deleted or supplemented *atu4545* exhibited similar growth characteristics on common and phenolic acid-containing carbon sources as strains with deleted or supplemented *atu4544*. Strains with a *pobA::lacZ* reporter fusion showed that PobR induced *pobA* expression. In addition, the use of a *pobR::lacZ* reporter fusion showed that PobR represses its expression. Electromobility shift assay revealed that the PobR regulator can bind specifically to DNA. The binding site was identified as CGTGCGATGGTGGATT. Deletions of *atu4544* (*pobA*) and *atu4545* (*pobR*) decreased *A. tumefaciens* pathogenicity by infecting carrot roots and kalanchoe leaves, with no effect on *virB* genes, and decreased bacterial biomass when phenolic acids were present. The collective findings demonstrate how transcriptional regulation by *A. tumefaciens* controls the metabolism of 4-hydroxybenzoate and imply that PobA and PobR aid in bacterial survival during host plant infection.

**IMPORTANCE:**

*Agrobacterium tumefaciens* is a widely distributed environmental bacterium and a recognized phytopathogen. Phenolic acids influence the relationship between *A. tumefaciens* and plants. One of the most important phenolic acids found in soil is 4-hydroxybenzoate, which is generated by plants. Mutants defective in the *atu4544* and *atu4545* genes inhibit *A. tumefaciens* tumor development. The *atu4544*-encoded enzyme, PobA, can metabolize 4-hydroxybenzoate, and the expression of its gene is positively regulated by the transcription factor encoded by *atu4545*. The *atu4545* gene is subject to negative autoregulation. The binding site of atu4545 is CGTGCGATGGTCGGATT. Dual regulation of regulators for phenolic acid catabolism may aid in the maintenance of appropriate quantities of phenolic compounds. These results clarify the pathogenic mechanisms of *A. tumefaciens* and broaden the understanding of the metabolic control mechanisms of phenolic chemicals.

## INTRODUCTION

*Agrobacterium tumefaciens* is a common gram-negative plant pathogen that is prevalent in the soil, on plant surfaces, and within plants. In the natural environment, *A. tumefaciens* infects wounded tissues of dicotyledonous plants, forming crown galls (1). The pathogenicity of *A. tumefaciens* relies on a specific plasmid termed the tumor-inducing (Ti) plasmid (2). In addition, virulence genes located on the chromosome can affect virulence gene expression in *A. tumefaciens*. For example, *chvA* and *chvB* primarily aid in attaching *A. tumefaciens* to host plants (3). The Ti plasmid is crucial for *A. tumefaciens* pathogenicity as it facilitates the introduction of T-DNA into the host genome (4). The transfer of T-DNA from *A. tumefaciens* to host cells is initiated by the activation of virulence (*vir*) genes in response to plant compounds and signals (5). Acetosyringone and α-hydroxy acetosyringone, which are found exclusively in the exudates of injured plant cells, are commonly used inducers of *vir* gene expression (6). Other phenols and phenolic acids, such as syringic acid, ferulic acid, and vanillic acid, can affect the interactions between *A. tumefaciens* and its host plants (7).

Phenolic acids, such as ferulic acid, vanillic acid, 4-hydroxybenzoate, and protocatechuate, are generated through lignin degradation and secretion by plant cells and are often found in the proximity of plant roots (8, 9). The catabolism of phenols and phenolic acids is mostly dependent on the β-ketoadipate pathway, which is found in fungi and soil bacteria (10). The β-ketoadipate pathway comprises two branches, the protocatechuate and catechol routes. Both branches lead to the production of the same metabolic product, 3-oxoadipate enol-lactone (11). Among the various aromatic compounds, 4-hydroxybenzoate is particularly common (12) and is converted to protocatechuate by the microbial hydroxylase encoded by *pobA*. The degradation pathway of 4-hydroxybenzoate is associated with the pathogenicity of phytopathogens, such as *Xanthomonas campestris* (*Xcc*) (13), *Fusarium oxysporum* f. sp. *lycopersici* (14), and *Cochliobolus heterostrophus* (15). The lesion length of *Xcc* mutant strains Δ*pobA* and Δ*pcaGH* is shorter compared to wild-type *Xcc* in Chinese radish. Invasion of tomato roots was reportedly impaired in a *cmle* (3-carboxy-cis, cis-muconate lactonizing enzyme) deletion mutant of *F. oxysporum* f. sp. *lycopersici*. In addition, β-ketoadipate pathway genes in *C. heterostrophus* help to detoxify plant phenolic compounds, thus preventing cell death of the fungal pathogen.

**Fig 1:**
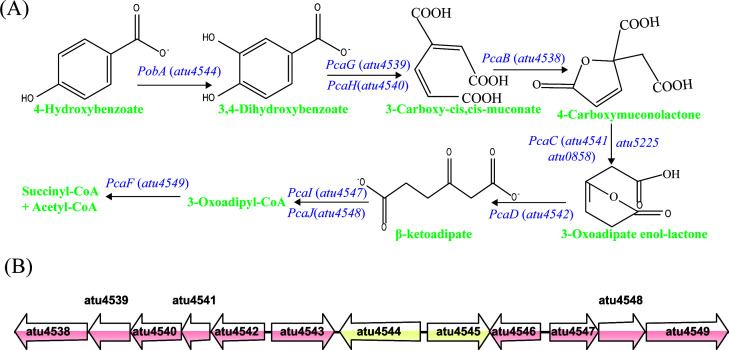
4-Hydroxybenzoate degradation pathway in *A. tumefaciens*. (**A**) Proposed 4HBA metabolic pathway. (**B**) Genetic map of *pca* and *pob* genes in *A. tumefaciens*. *pcaB*(*atu4538*), *pcaG*(*atu4539*), *pcaH*(*atu4540*), *pcaC*(*atu4541*), *pcaD*(*atu4542*), *pcaQ*(*atu4543*), *pobA*(*atu4544*), *pobR*(*atu4545*), *pcaR*(*atu4546*), *pcaI*(*atu4547*), *pcaJ*(*atu4548*), and *pca*F(*atu4549*).

The effect of 4-hydroxybenzoate degradation pathway on microbial pathogenicity in *A. tumefaciens* remains unclear. The *atu4544* (*pobA*) gene encodes 4-hydroxybenzoate hydroxylase, which is involved in the degradation of 4-hydroxybenzoate in the β-ketoadipate pathway. This chromosomally encoded convergent pathway degrades aromatic compounds (10). The pathway is composed of the *pob* and *pca* genes organized in a superoperon in *A. tumefaciens*. Genomic research has revealed the degradation of phenolic acids via protocatechuate routes in *A. tumefaciens* C58 (Fig. 1). The *pca* structural genes, organized in two distinct operons, *pcaBGHCD* (*atu4542-atu4538*) and *pcaIJF* (*atu4547--atu4549*), which are approximately 4 kb apart, are involved in protocatechuate degradation. The structural genes *pcaBGHCD* encode β-ketoadipate enol-lactone hydrolase, γ-carboxymuconolactone decarboxylase, protocatechuate 3,4-dioxygenase, and β-carboxy-*cis*,*cis*-muconate lactonizing enzyme. The *pcaIJ* genes encode β-ketoadipate succinyl-coenzyme A transferase. The *pcaF* gene encodes β-ketoadipyl CoA thiolase. These genes have been studied by growth phenotypes of knockout mutants and enzyme activities (16). Three regulatory genes were predicted in the β-ketoadipate pathway, that is, *atu4543* (*pcaQ*), *atu4545* (*pobR*), and *atu4546* (*pcaR*). LysR-type regulatory protein PcaQ (Atu4543) regulates the *pcaBGHCD* operon (17). Expression of the *pcaIJ* genes was not affected in the pcaQ: Ω background of strains and was induced by the coeffector β-ketoadipate (16). The *pcaIJF* genes may be regulated by a LysR-type regulatory protein PcaR (atu4546) based on amino acid sequence homology. Transcriptional regulation of 4-hydroxybenzoate hydroxylase (PobA) is mediated by PobR, which belongs to the AraC family of transcriptional regulatory proteins evolutionarily distant from the other two regulators in the aromatic compound-degradative pathways (16, 17). The operon organization and regulation of the *pob-pca* genes in *A. tumefaciens* differ from those of *Acinetobacter calcoaceticus* and *Pseudomonas putida* (18). These differences may reflect the ability of these species to adapt to various ecological niches and selection pressures. The molecular genetic characterization of the degradation of 4-hydroxybenzoate in *A. tumefaciens* provides an excellent model for determining the evolutionary process of catabolic pathways.

This study investigated the role of the metabolic gene *pobA* (*atu4544*) in the degradation of phenolic acids. Upstream of *atu4544,* there is a gene, *atu4545*, which encodes a transcription factor. This transcription factor was found to have dual regulation properties. The effects of *atu4544* and *atu4545* on the tumorigenicity of *A. tumefaciens* were assessed, and a preliminary investigation assessed the pathogenic mechanism. The study findings expand previous research on the β-ketoadipate pathway in *A. tumefaciens* and open up new possibilities for understanding the pathophysiology of *A. tumefaciens*.

## RESULTS

### Function of *A. tumefaciens atu4544*

**Fig 2:**
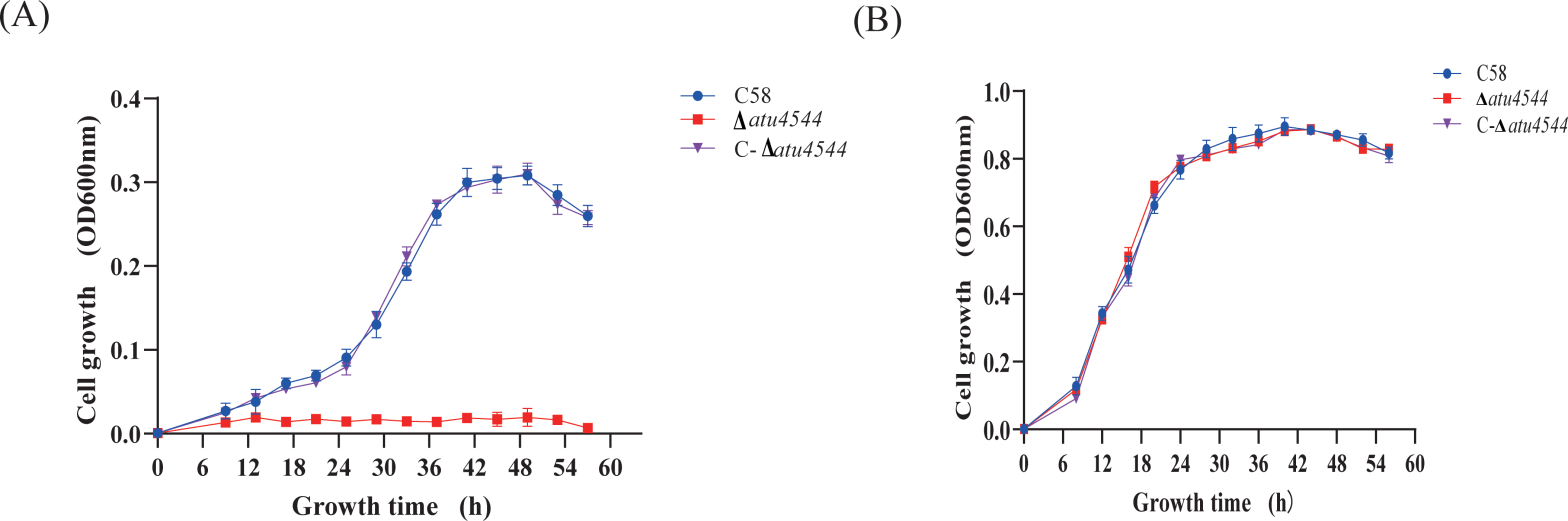
Cell growth of *A. tumefaciens* strains (C58, Δ*atu4544*, and C-Δ*atu4544*) on AB minimal medium with phenolic acids as carbon sources. 5 mM 4-hydroxybenzoate (**A**) and 10 mM protocatechuate (**B**) as the carbon source. Every point represents a standard deviation from the mean of a minimum of three replicates.

Of the variety of aromatic compounds found in soil and produced by plants, 4-hydroxybenzoate is one of the most common (12). This enzyme, encoded by *pobA,* catalyzes the hydroxylation of the C3 carbon atom of 4-hydroxybenzoate on the benzene ring, converting it to protocatechuate. Subsequently, protocatechuate is metabolized through the β-ketoadipate pathway (Fig. 1). In *A. tumefaciens* C58, the *atu4544* gene on the linear chromosome was designated to encode 4-hydroxybenzoate 3-monooxygenase. To study the *atu4544* gene and its encoded protein, an *atu4544* knockout strain was created by homologous recombination with the suicide plasmid pEX18Km. The complementation strain designated C-Δ*atu4544* was obtained by expressing *atu4544* in the strain Δ*atu4544* using the plasmid pUCA19. Cell growth of the wild-type C58, knockout mutant, and the complementation strain C-Δ*atu4544* was investigated on common sugars (sucrose and arabinose) and phenolic acids (4-hydroxybenzoate and protocatechuate) as the sole carbon source. As shown in Fig. S1, the three strains exhibited similar growth trends on common carbon sources, suggesting that *atu4544* had no influence on the central carbon metabolism of *A. tumefaciens. A. tumefaciens* C58 can utilize 4-hydroxybenzoate as the sole carbon source. When *atu4544* was deleted, 4-hydroxybenzoate was not utilized as a carbon source. Growth deficiency was restored when *atu4544* was expressed in Δ*atu4544* (Fig. 2A). When protocatechuate was used as the sole carbon source, *A. tumefaciens* C58, strain Δ*atu4544*, and C-Δ*atu4544* showed comparable growth trends, and their biomass reached nearly OD_600_ of 1.0 at the stationary phase (Fig. 2B). Cell growth determined in the presence of phenolic acids indicated the involvement of *atu4544* in 4-hydroxybenzoate catabolism, rather than protocatechuate metabolism, in the β-ketoadipate pathway.

To determine whether *atu4544* encoded 4-hydroxybenzoate 3-monooxygenase, its protein expression and enzymatic activity were examined. The *atu4544* nucleotide sequence was used to construct an isopropyl β-D-1-thiogalactopyranoside (IPTG)-inducible clone in *Escherichia coli* BL21 with plasmid pGex-4t-1, and the glutathione (GST)-tagged recombinant protein was extracted using an affinity chromatography resin. Figure S2A shows the expression of GST-atu4544, which has a molecular mass of approximately 70 kDa. The empty plasmid pGEX-4T1 was also purified (Fig. S2B) and used for enzymatic activity assays. The retention periods in the high-performance liquid chromatography (HPLC) trace (Fig. S3A and B) of 17.3 and 8.4 min for 4-hydroxybenzoate and protocatechuate, respectively, indicated that the mobile phase utilized is capable of effectively separating 4-hydroxybenzoate and protocatechuate. Only 4-hydroxybenzoate was observed when the purified GST-tag protein was introduced; no protocatechuate was produced (Fig. S3C). The peak area of 4-hydroxybenzoate as the substrate was reduced with the addition of purified atu4544 protein and NADPH (or NADH) using the flavin cofactor FAD, and the concentration decreased similarly (Fig. S3D and E). The peak area and concentration of protocatechuate progressively increased as the enzymatic reaction proceeded (Fig. S3D and E). Atu4544 (PobA) catalyzed the hydroxylation of 4-hydroxybenzoate to produce protocatechuate. Enzyme activities of PobA were 1.96 and 4.53 U/mg when NADH and NADPH, respectively, were used as the electron donor for the enzymatic assay.

### Regulatory role of *A. tumefaciens atu4545*

**Fig 3:**
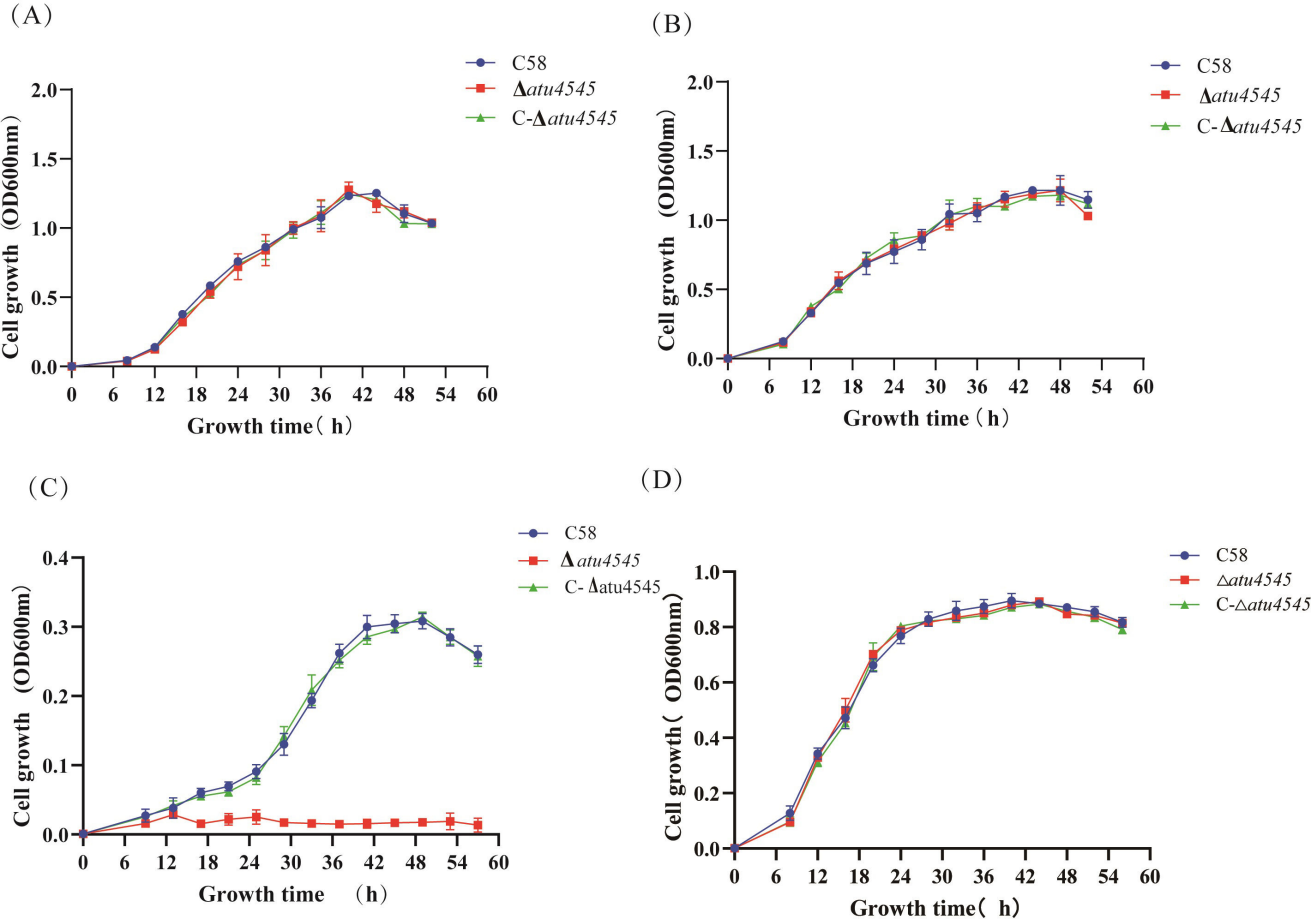
Cell growth of *A. tumefaciens* strains (C58, Δ*atu4545*, and C-Δ*atu4545*) on AB minimal medium with different sole carbon sources. (**A**) 15 mM sucrose as the sole carbon source, (**B**) 15 mM arabinose as the sole carbon source, (**C**) 5 mM 4-hydroxybenzoate as the sole carbon source, and (**D**) 10 mM protocatechuate as the sole carbon source. Every point represents a standard deviation from the mean of a minimum of three replicates.

**Fig 4:**
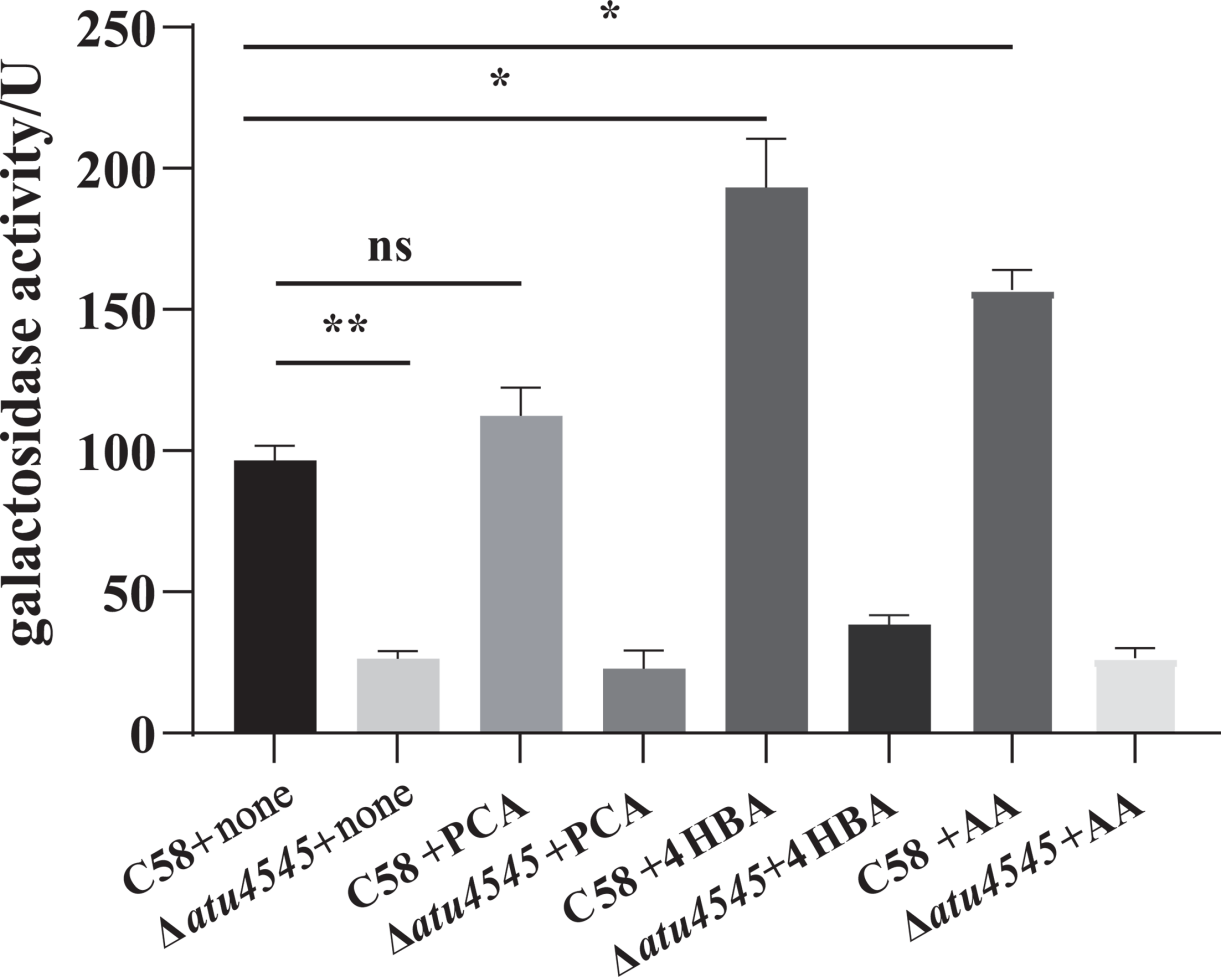
Effects of *atu4545* on *atu4544* promoter activity using β-galactosidase activity. C58 and Δ*atu4545* correspond to *A. tumefaciens atu4544-lacZ* and *A. tumefaciens* Δ*atu4545* atu*4544-lacZ*. They were grown in AB-arabinose (Ara) medium. Protocatechuate (PCA), 4-hydroxybenzoate (4HBA), and adipic acid (AA) were, respectively, added to the Ara medium. Data are presented as averages of three independent experiments. The error bars indicate the standard deviation of the mean. **P < 0.01; *P < 0.05; n.s., not significant.

Regulation of *pobA* expression by the transcription factor PobR has been documented in several bacterial species, including *A. calcoaceticus*, *Azotobacter chroococcum*, *Cupriavidus necator*, *Streptomyces coelicolor*, and *Xanthomonas campestris* (19–23). In *A. calcoaceticus*, the transcriptional activator PcaU shares structural similarities with PobR (24). In *P. putida*, both PobC and PobR regulate *pobA* expression (25). In *A. tumefaciens* C58, the gene *atu4545* (*pobR*) is located upstream of *atu4544* and has been annotated as an AraC family transcriptional activator. The strains Δ*atu4545* and C-Δ*atu4545* were constructed and cultivated on common carbon sources and phenolic acids. The growth trends of these *atu4545* mutants (Fig. 3) were consistent with the *atu4544* mutants on certain carbon sources, indicating a correlation between *atu4544* and *atu4545*. To investigate the *atu4545*-encoded transcription factor that may act on *atu4544* expression, an *atu4544-lacZ* fusion on the *A. tumefaciens* chromosome was created to represent *atu4544* expression by detecting β-galactosidase activity. Theoretically, the function of *atu4544* is not affected by *lacZ* insertion because the *lacZ* gene is linked after the stop codons of *atu4544* by a strong ribosome-binding site sequence. Two recombinant strains, *A. tumefaciens atu4544-lacZ* and *A. tumefaciens* Δ*atu4545 atu4544-lacZ*, respectively, displayed similar growth phenotypes to the wild-type and *A. tumefaciens* Δ*atu4545* (Fig. S4). Subsequently, β-galactosidase activities of the two recombinant strains were measured on an AB-arabinose mineral medium (Fig. 4). Compared with *A. tummefaciens atu4544-lacZ*, β-galactosidase activity of *A. tummefaciens* Δ*atu4545 atu4544-lacZ* decreased significantly by 74%, indicating that the *atu4544* expression could be triggered by the transcription factor atu4545. The effect of external substances on the expression of *atu4544* was studied. 4-hydroxybenzoate, protocatechuate, or adipic acid was tested as PobR effectors in AB-arabinose growth medium based on earlier studies of *pobR* homologous genes in other bacterial species and metabolites in the β-ketoadipate pathway in *A. tumefaciens*. Adipic acid, as a nonmetabolizable analog of β-ketoadipate, was added into arabinose medium to investigate the enzyme activities of the β-ketoadipate pathway in *A. tumefaciens* (26). Figure 4 shows that the addition of 4-hydroxybenzoate, protocatechuate, and adipic acid boosted *atu4544* expression by 2.01, 1.17, and 1.64 times, respectively. 4-Hydroxybenzoate and adipic acid had the potential to greatly increase *lacZ* expression (*P* < 0.05). These phenotypic studies and enzyme activities of related mutants revealed that atu4545 positively regulated the expression of *atu4544* and that 4-hydroxybenzoate and adipic acid can increase its regulatory function more than protocatechuate as inducers.

**Fig 5:**
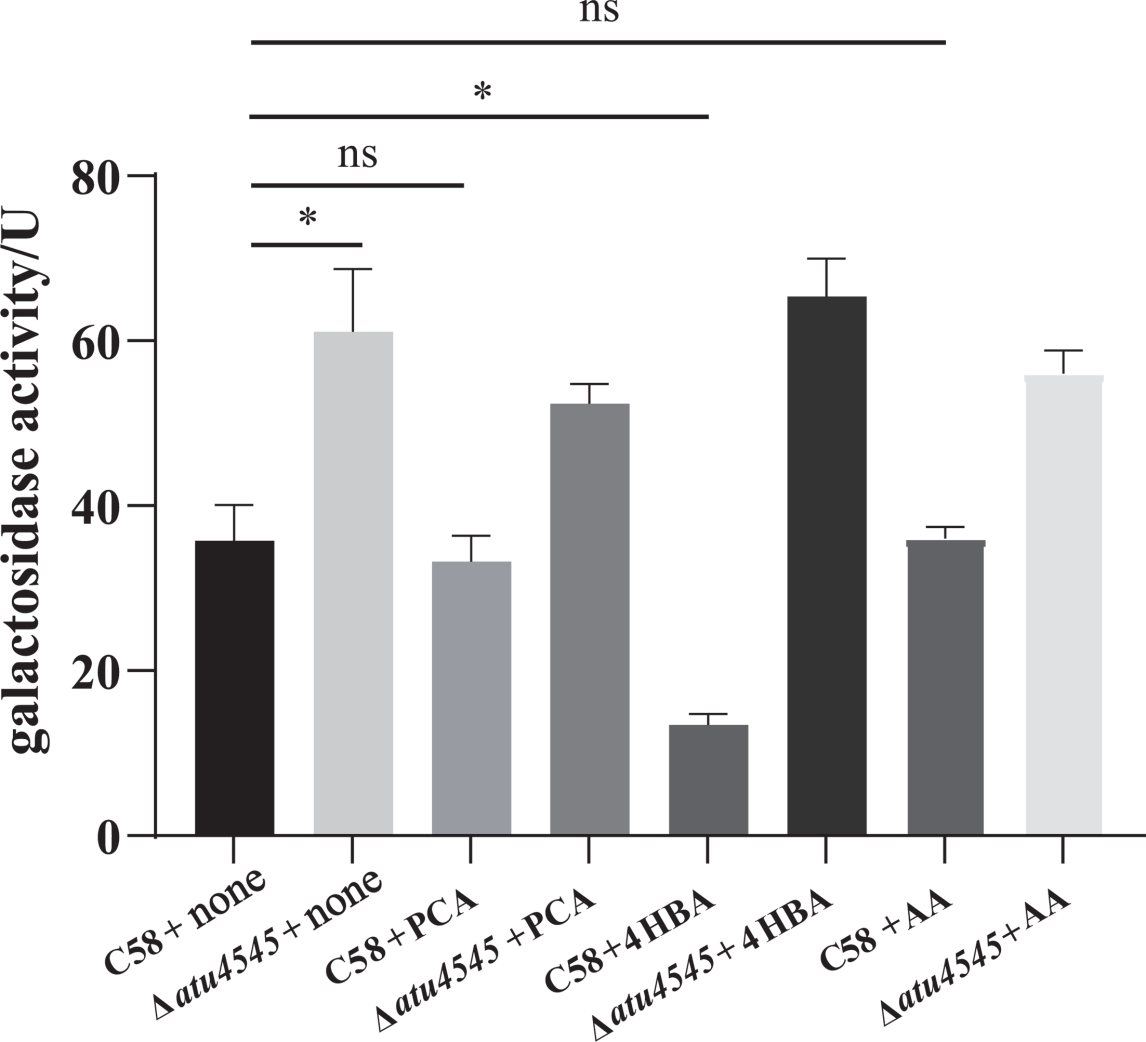
Effects of atu4545 on *atu4545* promoter activity using β-galactosidase activity. C58 and Δ*atu4545* correspond to *A. tumefaciens lacZ* and *A. tumefaciens* Δ*atu4545 lacZ*, and they were grown in AB-arabinose (Ara) medium. Protocatechuate (PCA), 4-hydroxybenzoate (4HBA), and adipic acid (AA) were, respectively, added to Ara medium. Data shown are the average of three independent experiments. The error bars indicate the standard deviations of the mean. *P < 0.05; n.s., not significant.

Autoregulation of PobR has been documented in *A. calcoaceticus* (27) and *X. campestris* (23). The *atu4545* promoter was ligated to the *lacZ* gene using plasmid pCB301 to examine whether *atu4545* influences its own expression in *A. tumefaciens*. The pCB301 P*pobR-lacZ* was transformed into *A. tumefaciens* C58 and *A. tumefaciens* Δ*atu4545* to obtain *A. tumefaciens lacZ* and *A. tumefaciens* Δ*atu4545 lacZ*, respectively. P*pobR*-dependent β-galactosidase activities in the strain Δ*atu4545 lacZ* were all higher than those in *A. tumefaciens lacZ* under the same condition (Fig. 5). On AB-arabinose minimum medium, the β-galactosidase activity of strain Δ*atu4545* was 1.44 times higher than that of *A. tumefaciens lacZ*. The enhanced promoter activity of *atu4545* following its deletion indicated that the expression of *pobR* was negatively controlled by its own gene products. After adding 4-hydroxybenzoate, protocatechuate, or adipic acid, the ratio of lacZ activity in *A. tumefaciens* Δ*atu4545 lacZ* to lacZ activity in *A. tumefaciens lacZ* was 3.14, 0.99, and 0.98 times, respectively, of the lacZ activity ratio grown on AB-arabinose minimum medium. 4-Hydroxybenzoate enhanced the self-regulation of PobR compared to protocatechuate and adipic acid. There was no significant increase in lacZ activity after adding protocatechuate and adipic acid to strain Δ*atu4545*, which indicated that protocatechuate and adipic acid had no obvious effect on enhancing *pobR* gene self-regulation. 4-Hydroxybenzoate, rather than protocatechuate or adipic acid, enhanced the autoregulation of the *pobR* gene.

### PobR protein binds directly to the intergenic region of *atu4544* and *atu4545*

**Fig 6:**
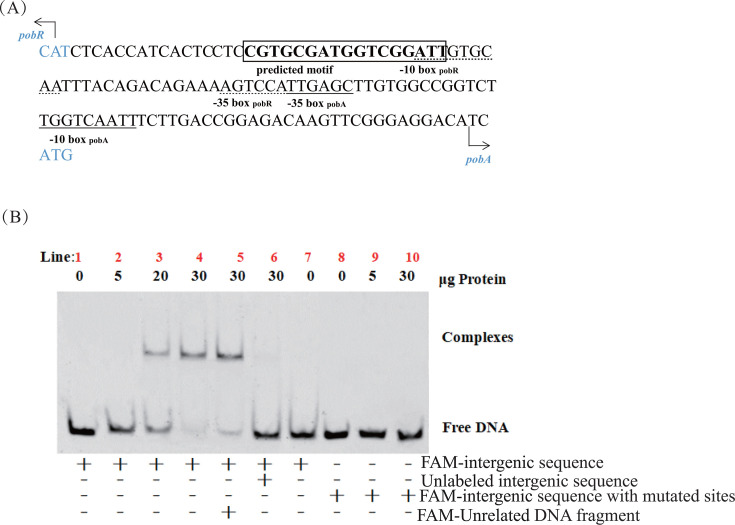
PobR protein binds to the intergenic region of *atu4544* and *atu4545*. (**A**) Predicted overlapping promoters between *atu4544* and *atu4545*. The −35 and −10 elements of the promoter were labeled and predicted by the BPROM online tool. The sequences in the box are possible binding sites. Two arrows represent the start of the respective ORFs. (**B**) EMSA results of PobR protein with the FAM-labeled DNA. Unlabeled intergenic sequences or mutated binding sites or unrelated DNA were added to determine the specificity of the binding. BSA protein was only added in lane 7 as a negative control.

Genome analysis allowed predicting that the intergenic region of *atu4544* and *atu4545* had two divergent promoters. Transcription factor binding sites were predicted using the MEME suite (28) (Fig. 6A). To test whether atu4545 can bind to the intergenic region of *atu4544* and *atu4545*, an electrophoretic mobility shift assay (EMSA) was performed (Fig. 6B). The intergenic regions were labeled with FAM oligonucleotides. The labeled intergenic region did not form shifted bands with the bovine albumin protein used as a negative control (lane 7). When the concentration of purified His-atu4545 protein (Fig. S5) was increased to 20 and 30 µg, the incubation of the labeled intergenic region and the purified atu4545 protein formed retarded bands (lanes 2–5). These shifts indicated the interaction between the atu4545 protein and natural DNA probes, which were not influenced by unrelated DNA fragments (lane 5). The possible binding motifs were validated by replacing the motif with the internal sequences of *atu4544* of the same length (17 bp). The purified atu4544 did not form retarded bands (lanes 8–10) with the mutated DNA probe. The purified PobR protein was precisely attached to the upstream sequences of *atu4544* and *atu4545*; the atu4545-binding sites were CGTGCGATGGTCGGATT, according to the EMSA results of the original and modified DNA probes. In addition, EMSA was conducted using varying concentrations of 4-hydroxybenzoate and adipic acid. After the addition of 4-hydroxybenzoate, the shifted band intensity strengthened, indicating that a given mass of 4-hydroxybenzoate (0.25, 0.5, and 1.0 mM) increased the formation of the PobR/DNA probe complex (Fig. S6A). When a small amount of adipic acid was added, the shifted band remained almost unchanged. The increased adipic acid seemed to hinder the binding of the PobR/DNA probe (Fig. S6B).

### Deletion of *atu4544*/*atu4545* impairs the infection of host plants

**Fig 7:**
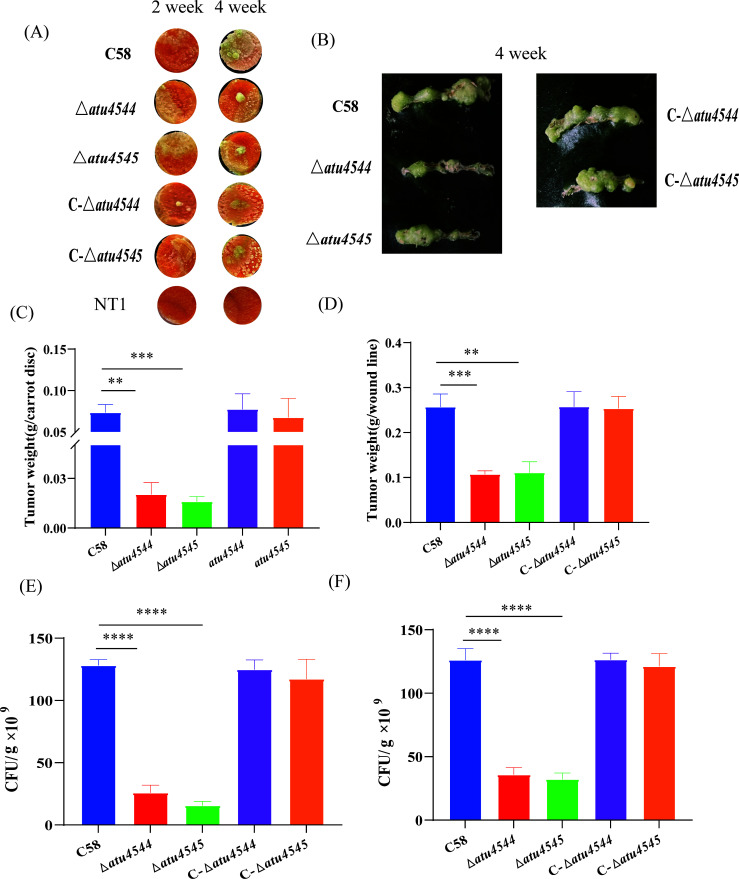
Effects of *atu4544* and *atu4545* on tumorigenesis of *A. tumefaciens*. (**A**) Tumorigenesis of *A. tumefaciens* strains on carrot root discs. (**B**) Tumorigenesis of *A. tumefaciens* strains on kalanchoe leaves. (**C**) Tumor weights after infecting the carrots for 4 weeks. (**D**) Tumor weights after infecting kalanchoe for 4 weeks. (**E**) The number of colonies in tumors on carrots infected by *A. tumefaciens* strains after 4 weeks. (**F**) The number of colonies in tumors from kalanchoe leaves infected by *A. tumefaciens* strains after 4 weeks. * denotes significant difference; ***P < 0.001; ****P < 0.0001.

Effects of *atu4544*/*atu4545* on plant infection were evaluated by measuring tumorigenesis on carrot root discs and kalanchoe leaves (Fig. 7A and B). The inoculated strains were *A. tumefaciens* NT1, C58, Δ*atu4544,* C-Δ*atu4544*, *Δatu4545,* and C-Δ*atu4545*. After 4 weeks, the tumors were scraped and weighed for statistical analysis. On the carrot plates, the NT1 strain lacking the Ti plasmid was almost completely non-tumorigenic. The tumor weighing results showed that the deletion of *atu4544* and *atu4545* resulted in the tumor weight reduction of 74% and 76% on carrot root discs, and 62% and 59% on kalanchoe leaves, respectively. Tumor weights of C-Δ*atu4544* and C-Δ*atu4545* could approach 100% and 94% of the wild type, respectively, indicating that gene expression of *atu4544*/*atu4545* could regain the tumorigenesis of the respective knockout mutants (Fig. 7C and D). Bacteria collected from different tumors were diluted and counted in AB-sucrose medium. The number of colonies per 0.1 g tumor in Δ*atu4544* and Δ*atu4545* was only 20% and 6% for carrot root discs and 32% and 21% for kalanchoe leaves, respectively, of those in *A. tumefaciens* C58 (Fig. 7E and F). The reduction in the number of colonies in tumors suggests that the loss of *atu4544*/*atu4545* affects the survival and growth of *A. tumefaciens* in infected plants. 4-hydroxybenzoate concentrations in carrot root discs were assessed using tumors that had grown after 2, 3, or 4 weeks. Water and methanol extraction did not detect any 4HBA. To determine 4HBA concentrations in tumors, heterologous 4-hydroxybenzoate was added and quantified. Tumor weights (Fig. S7A) and colony development (Fig. S7B) in the tumors of the strains were comparable to those without exogenous 4HBA. The loss of *atu4544*/*atu4545* resulted in a more than twofold increase in 4HBA levels relative to the wild-type C58 (Fig. S7C). Following supplementation with *atu4544*/*atu4545*, the 4HBA levels decreased considerably, indicating that *A. tumefaciens*’ 4HBA metabolic ability was restored. Both tumor weight and colony development in tumors showed that deletion of *atu4544*/*atu4545* reduced *A. tumefaciens* pathogenicity.

### *pobA*/*pobR* affects pathogenicity, rather than *virB* gene expression, of *A. tumefaciens* by affecting growth

**Fig 8:**
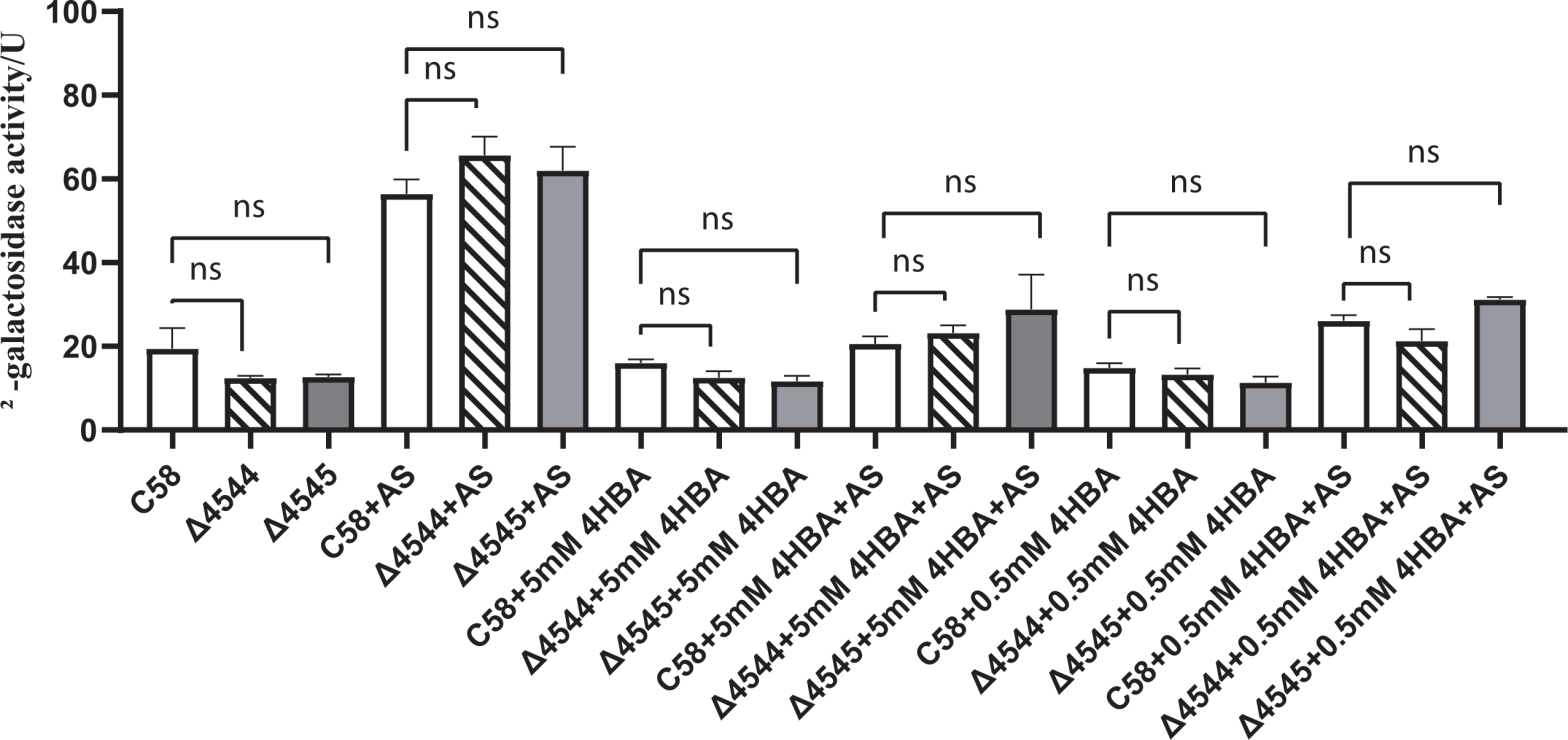
Effects on *virB* gene expression of *atu4544* and *atu4545* in *A. tumefaciens*. Strains used here were engineered based on the *virB-lacZ* reporter fusion strain (*A. tumefaciens virB-lacZ*). AS denotes acetosyringone (100 µM), and 4HBA (5 mM and 0.5 mM) denotes 4-hydroxybenzoate. “ns” means no significance.

**Fig 9:**
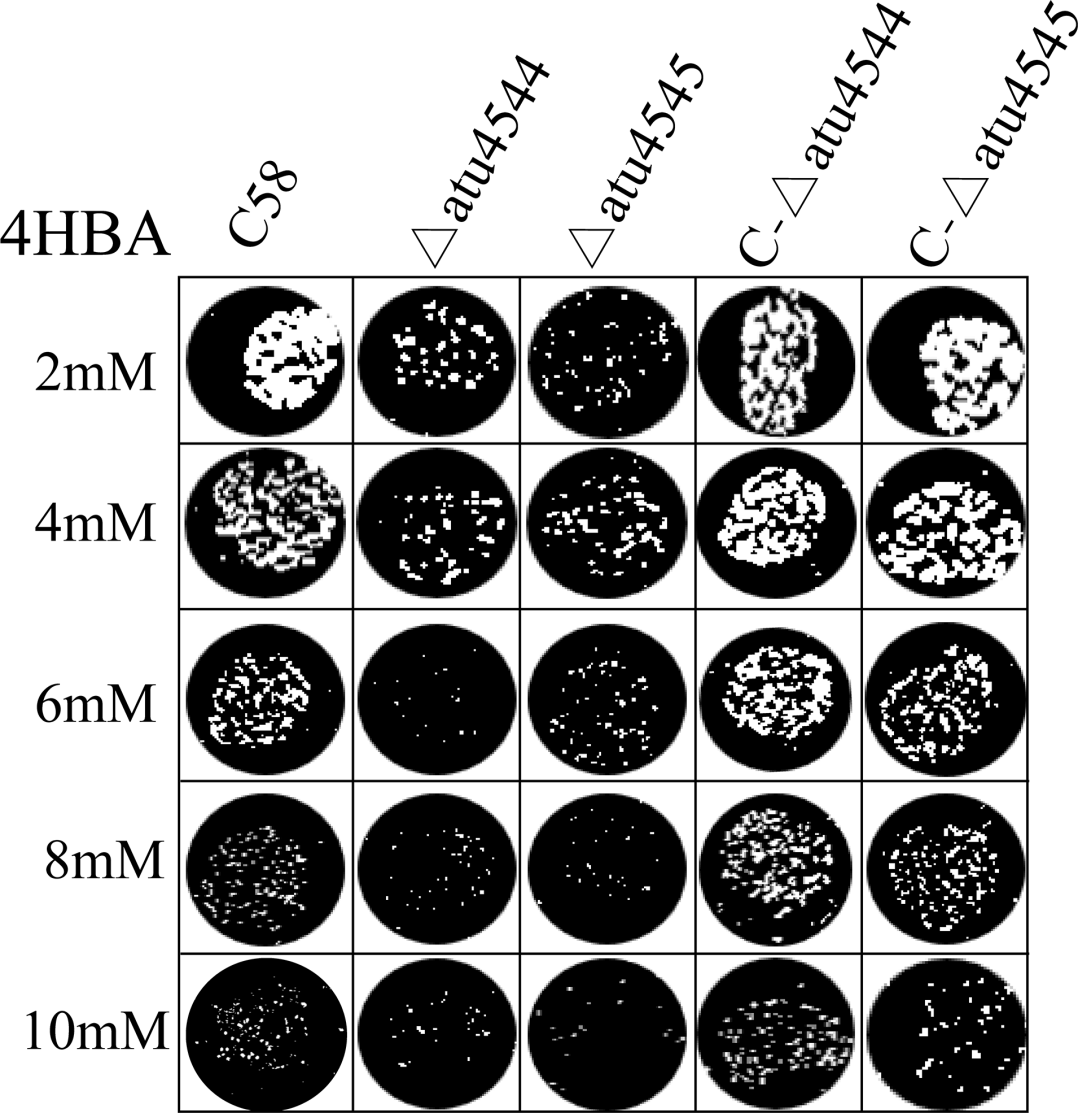
Pot assays of *A. tumefaciens* growth on plates with different 4-hydroxybenzoate concentrations.

**Fig 10:**
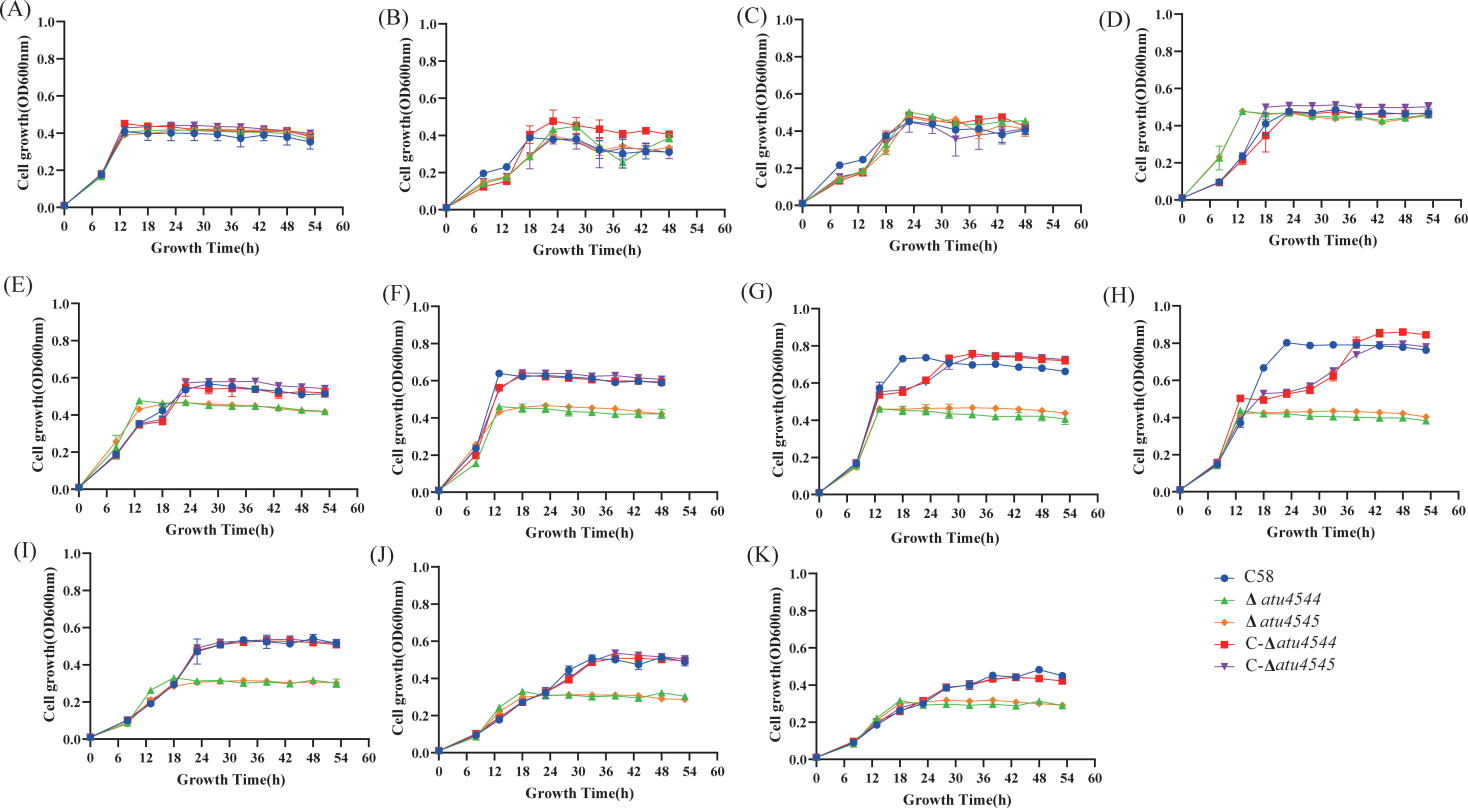
Growth curves of *A. tumefaciens* at different 4-hydroxybenzoate concentrations on 0.05% AB-sucrose medium. (**A**) no 4HBA, (**B**) 0.02 mM 4HBA, (**C**) 0.2 mM 4HBA, (**D**) 2 mM 4HBA, (**E**) 4 mM 4HBA, (**F**) 6 mM 4HBA, (**G**) 8 mM 4HBA, (**H**) 10 mM 4HBA, (**I**) 12 mM 4HBA, (**J**) 14 mM 4HBA, and (**K**) 16 mM 4HBA.

Effects of *atu4544*/*atu4545* on *virB* expression were first evaluated in *A. tumefaciens*. The previously constructed *A. tumefaciens virB-lacZ* as–chassis strains, *atu4544* and *atu4545*, were individually deleted. The activity of β-galactosidase was measured to determine whether the two gene knockouts would change the expression of the *virB* gene. As shown in Fig. 8, acetosyringone increased the relative expression of the *virB* gene in *A. tumefaciens*. The *atu4544* and *atu4545* gene deletion strains did not significantly differ from that of wild-type C58 when acetosyringone was added, suggesting that *atu4544* and *atu4545* genes did not affect the expression of *virB*. The addition of 4-hydroxybenzoate significantly reduced the expression of the *virB* gene. Low β-galactosidase activity in the presence of 4-hydroxybenzoate was linked to cellular states suppressed by phenolic compounds. For example, vanillic acid inhibits *virB* gene expression (7). The wild-type C58, Δ*atu4544*, and Δ*atu4545* strains still showed no difference in *virB* gene expression when 4-hydroxybenzoate was present. The underlying reason for *atu4544*/*atu4545* influencing the host plant infection is thought to affect the cell growth of *A. tumefaciens*. Pot assays of *A. tumefaciens* strains on plates (Fig. 9) showed that cell growth was inhibited with increasing 4-hydroxybenzoate concentrations. At the same concentration of 4-hydroxybenzoate, the colonies of the knockout mutants were fewer than those of the wild-type and complemented strains. Similar trends were also seen in growth curves on liquid medium (Fig. 10). Lower colony development in tumors of *atu4544*/*atu4545* mutant tumors was linked to the decreased *A. tumefaciens* growth. Decreased tumorigenesis in host plants could be related to the decrease in bacterial proliferation caused by the absence of *atu4544/atu4545*.

## DISCUSSION

In addition to growing independently, *A. tumefaciens* is a plant pathogen that coexists with plants. During normal growth and development, plants produce a wide range of phenolic compounds in both root and shoot tissues (29). The β-ketoadipate pathway breaks down 4-hydroxybenzoate and other phenolic chemicals (30). The enzymes of the β-ketoadipate pathway are conserved among bacteria, but their regulation and organization have evolved significantly (10)(31). The study of *atu4544* and *atu4545* extends the knowledge of the β-ketoadipate pathway in *A. tumefaciens*. The β-ketoadipate pathway in *A. tumefaciens* includes *atu4543* (*pcaQ*), *atu4545* (*pobR*), and *atu4546* (*pcaR*). The LysR regulatory protein PcaQ has been validated as an activator of *pcaBGHCD* (17). The study of the other two regulatory genes depended only on BLAST analysis. *atu4546* shared 42.7% amino acid sequence identity with PcaR of *P. putida. The atu4545* gene is homologous to a putative *pobR* gene in *Rhizobium leguminsarum* biovar; however, it does not show homology with PobR of *A. calcoaceticus* (18). The ability of *atu4544* (*pobA*) to catalyze the conversion of 4-hydroxybenzoate to protocatechuate was examined in this study, as it was essential for cell growth on AB-4-hydroxybenzoate minimum medium. The AraC regulatory gene, *atu4545* (*pobR*), was predicted to be located on the nucleotide sequence upstream of *atu4544*. The ability of PobR to bind to the promoter site CGTGCGATGGTCGGATT and activate *atu4544* expression was confirmed.

The β-ketoadipate pathway of *A. tumefaciens* was similar to the most thoroughly studied bacterial species (*P. putida and A. calcoaceticus)*, but had its distinctive patterns of transcriptional control (32). Similar to many gram-negative bacteria, the *pobA* gene is linked to the *pobR* gene in *A. tumefaciens*. The intergenic region between *pobA* and *pobR*, a bidirectional promoter, drives coordinated transcription in opposite directions. The regulatory role of PobR in the *pobA* gene can be divided into positive and negative. The combination of *in situ* knockout and reporter gene assay suggests that atu4545 activates the expression of *atu4544*. Despite the differences in protein sequence similarity, for example, *A. chroococcum* PobR has no homology to the PobR protein in *Acinetobacter* strain ADP1, and positive regulation by PobR is currently found in most bacteria, such as *X. campestris* (23), *A. chroococcum* (20), *A. calcoaceticus* (27), and *C. necator* (21). *S. coelicolor* is an exception, where PobR (SCO3209) negatively regulates *pobA* transcription (22). In *P. putida* WCS358, the regulatory protein PobC, which has a low identity with other PobR proteins, activates *pobA* expression (25). In addition to controlling *pobA*, PobR was also confirmed to regulate its expression in *A. tumefaciens*. This function was previously observed only in *A. calcoaceticus* and not in other bacteria. Furthermore, the binding sequences were compared with those of other known regulatory motifs using TOMTOM. This low similarity indicated a distinct regulatory role of PobR in *A. tumefaciens* (Fig. S8). Divergence in transcriptional regulation mechanisms might represent evolutionary flexibility and nuanced features of niche adaptation. Bacteria use different inducers for the β-ketoadipate pathway. For example, protocatechuate activates all of the protocatechuate branch genes in *A. calcoaceticus* but not any of the pca genes in *Agrobacterium* (10). In *Pseudomonas cepacia*, β-ketoadipate and 4-hydroxybenzoate induce *pcaB* expression (33). 4-hydroxybenzoate could enhance *atu4544* expression for 4HBA degradation and also induce pobR negative autoregulation, which may be linked to maintaining appropriate levels of 4-hydroxybenzoate. Adipic acid, a nonmetabolizable analog of β-ketoadipate, increases *atu4544* expression, indicating that pathway intermediates may affect 4-hydroxybenzoate metabolism in *A. tumefaciens* (16). Bacterial species exhibit diverse physiological regulation due to the inducibility of β-ketoadipate pathway genes by different pathway intermediates.

Deletion of *atu4544* and its transcriptional activator atu4545 reduced tumor weight and bacterial colonies in tumors when the strains were incubated on carrot root discs and kalanchoe leaves. Similarly, strains Δ*pobR*/Δ*pobA* of *Xcc* exhibited compromised virulence in Chinese radish (13). Additional genes in the β-ketoadipate pathway were linked to the infection of host plants in *F. oxysporum* f. sp. *Lycopersici* (14) and *C. heterostrophus* (15). The underlying mechanism of *the* β-ketoadipate pathway on microbial pathogenicity remains unknown. Expression of virulence genes is thought to be important for the pathogenicity of *A. tumefaciens* C58 (34). The promoter activity of *vir* genes in the *atu4544*/*atu4545* knockout mutants was equivalent to that in the C58 strain, indicating that 4-hydroxybenzoate hydroxylation did not affect *vir* gene expression. The compromised virulence of *atu4544*/*atu4545* knockout mutants may be related to cell growth inhibition in the presence of 4-hydroxybenzoate in pot assays.

In addition to 4-hydroxybenzoate, cell growth is also inhibited by other aromatic compounds, such as *p*-coumaric acid, ferulic acid, and vanillic acid (7) (Fig. S9 and S10). Plant roots generate phenolic compounds and serve dual functions of repelling and attracting different organisms to the plant environment, which could promote rhizobial nodulation and agrobacterial pathogenicity (35). Appropriate quantities of phenolic compounds may be most favorable to the infection of *A. tumefaciens* in plant hosts (36). 4-Hydroxybenzoate is one of the most significant aromatic compounds formed from plant-derived molecules and is present in soil (12). *Atu4544* could convert 4-hydroxybenzoate to the structurally similar protocatechuate, which is subsequently metabolized to tricarboxylic acid intermediates via the β-ketoadipate pathway (10). *Atu4545* harbors 4-hydroxybenzoate binding sites that are conserved with PobR sites of *Xcc*, including H8, R15, H19, W21, R27, Q33, Y36, H67, and R134. These sites were required for 4-hydroxybenzoate binding and the degradation of 4-hydroxybenzoate (13). Simultaneously, the negative autoregulation of atu4545 allows self-modification of its level to control the breakdown of 4-hydroxybenzoate. Another regulatory gene in the β-ketoadipate pathway, *pcaQ* (*atu4543*), was autorepressed and served as an activator of *pcaBGHCD* (*atu4542-atu4538*) (17). The *atu4546* regulatory gene was validated by dual regulation (unpublished data). Regulators frequently regulate the expression of several genes by binding to transcription factor binding sites upstream of their target genes or transcription units (37). Several transcription factors act as activators and repressors. One basic example is transcription factors that bind to a single location in the intergenic region between divergently transcribed units and regulate each in a unique manner (38). The metabolic function and transcriptional regulation of 4-hydroxybenzoate metabolism were adapted to *A. tumefaciens* infection of the host plant.

## MATERIALS AND METHODS

### Strains, plasmids, and growth conditions

The bacterial strains and plasmids used in this study are listed in Table 1. The primers and synthetic DNA fragments are listed in Table S1. *E. coli* strains were grown in Luria-Bertani (LB) medium at 37°C (39). *A. tumefaciens* strains were grown in MG/L medium or *Agrobacterium* (AB) minimal medium at 28°C (40). The sole carbon sources added to the AB minimal medium were 5 mM 4-hydroxybenzoate, 10 mM protocatechuate, 15 mM sucrose, and 15 mM L-arabinose for *A. tumefaciens*. Cultivation of plasmid-bearing strains required the addition of 50 or 100 mg/L kanamycin, 100 mg/L ampicillin, and 100 mg/L carbenicillin.

**TABLE 1:** Strains and plasmids used in this study^*a*^

Strains and plasmids	Characteristics	Source
Strains		
*E. coli* DH5a	Host for DNA cloning	(41)
*E. coli* BL21 (DE3）	Expression vector host	(42)
*E. coli* Atu4544	BL21(DE3) strain transformed using pGEX::GST-*atu4544*	This study
*E. coli* Atu4545	BL21(DE3) strain transformed using pET30a::HIS -*atu4545*	This study
*A. tumefaciens* C58	Wild type, nopaline-type pTiC58 plasmid	(43)
*A. tumefaciens* NT1	Lack of the Ti plasmid and no production of HSL	Integrative Microbiology Research Center South China Agricultural University
*A. tumefaciens* Δ*atu4544*	*atu4544* gene deleted in *A. tumefaciens* C58	This study
*A. tumefaciens* Δ*atu4545*	*atu4545* gene deleted in *A. tumefaciens* C58	This study
*A. tumefaciens* C-Δ*atu4544*	Complementary strain obtained by transforming pUCA19-*atu4544* into Δ*atu4544* strain	This study
*A. tumefaciens* C-Δ*atu4545*	Complementary strain obtained by transforming pUCA19-*atu4545* into Δ*atu4545* strain	This study
*A. tumefaciens atu4544-lacZ*	*lacZ* gene inserted *in situ* at *atu4544* downstream on the chromosome of *A. tumefaciens* C58	This study
*A. tumefaciens* Δ*atu4545 atu4544-lacZ*	*lacZ* gene inserted *in situ* at *atu4544* downstream on the chromosome of *A. tumefaciens* Δ*atu4545*	This study
*A. tumefaciens lacZ*	pCB301:: promoter*_atu4545_*:: *_lacZ_* transformed into *A. tumefaciens* C58	This study
*A. tumefaciens*Δ*atu4545 lacZ*	pCB301:: promoter*_atu4545_*:: *_lacZ_* transformed into *A. tumefaciens* Δ*atu4545*	This study
*A. tumefaciens virB-lacZ*	*lacZ* gene inserted *in situ* at *virB* downstream (located at the *virB* initiation codon +162 bp) of *A. tumefaciens* C58	Constructed by our lab (unpublished)
*A. tumefaciens virB-lacZ* Δ*atu4544*	*atu4544* gene deleted in *A. tumefaciens virB-lacZ*	This study
*A. tumefaciens virB-lacZ* Δ*atu4545*	*atu4545* gene deleted in *A. tumefaciens virB-lacZ*	This study
Plasmids		
pEX18Km	Gene replacement vector carrying a counter selectable marker *sacB*, *oriT*, Km^R^	(44)
pEX18Km-*atu4544*	500 bp upstream and 500 bp downstream fragments of *atu4544* integrated into the suicide plasmid pEX18Km to delete the *atu4544* gene; Km^R^	This study
pEX18Km-*atu4545*	500 bp upstream and 631 bp downstream fragments of *atu4545* integrated into the suicide plasmid pEX18Km to delete the *atu4544* gene; Km^R^	This study
pEX18Km-*atu4544-lacZ*	Three fragments integrated into the suicide plasmid pEX18Km to insert *lacZ* gene behind *atu4544* on the chromosome; Km^R^	This study
pUCA19	pUC19 carrying an agrobacterial replicon; Ap^R^, Cr^R^	(44)
pUCA19-*atu4544*	Plasmid pUCA19 carrying encoding sequence of *atu4544*; Ap^R^, Cr^R^	This study
pUCA19-*atu4545*	Plasmid pUCA19 carrying encoding sequence of *atu4545*; Ap^R^, Cr^R^	This study
pCB301	Minimum binary vector plasmid; Km^R^	(45)
pCB301:: promoter*_atu4545_*:: *_lacZ_*	Upstream of *atu4545* ligated to the *lacZ* gene integrated into pCB301	This study
pET30a	Protein-expressing plasmid; Km^R^	Novagen
pET30a:: HIS-*atu4545*	N-terminal HIS-tag of the pET30a ligated with 924 bp *atu4545* ORF; Km^R^	This study
pGEX-4T-1	Protein-expressing plasmid; Ap^r^, Cr^r^	GE Healthcare
pGEX::GST-*atu4544*	N-terminal GST-tag of the pGEX-4T-1 ligated with 1,173 bp *atu4544* ORF; Ap^r^, Cr^r^	This study

^*a*^ Km: kanamycin; Ap: ampicillin; Cr: carbenicillin.

### Construction of *A. tumefaciens* deletion mutants and complementary strains

*Atu4544* and *atu4545* were deleted in both *A. tumefaciens* C58 and *A. tumefaciens virB-lacZ*. The upstream and downstream sequences of the two genes were amplified using *Gene*-U-F/R and *Gene*-D-F/R primers (see Table S1). The gene knockout cassette was produced by overlapping PCR with *Gene*-U-F and *Gene*-D-R primers and detected by 1% agarose gel electrophoresis. Purified PCR products obtained using an agarose gel DNA extraction kit (TaKaRa Bio, Shiga, Japan) and the pEX18Km enzyme-digested suicide vector were ligated to convert *E. coli* DH5α. After PCR and DNA sequencing, the generated plasmids were transformed into *A. tumefaciens* C58 and *A. tumefaciens virB-lacZ*. After kanamycin resistance and sucrose sensitivity screening, gene deletion mutants were obtained and validated by DNA sequencing.

To construct the *atu4544* and *atu4545* expression plasmids, the coding sequences of these genes were amplified using C*-atu4544*-F/R and C*-atu4545*-F/R and inserted into the pUCA19 plasmid expression vector harboring the *lacZ* promoter. The correctly constructed plasmids were used to transform the corresponding deletion mutant strains of *A. tumefaciens*. *A. tumefaciens* C-*Δatu4544* and *A. tumefaciens* C-*Δatu4545* were verified by detecting kanamycin resistance and DNA sequencing.

### **Construction of** *A. tumefaciens* ***lacZ* reporter strains**

The *A. tumefaciens* strain harboring a genome containing the *in situ* inserted reporter gene *lacZ* was used to measure *atu4544* promoter activity in the presence or absence of *atu4545*. The *lacZ* gene was programmed to follow *atu4544* using the strong RBS sequence 5′-TTTCTCCTCTTT-3′ (46). The *lacZ* gene was amplified using the *atu4544-lacZ*-F/R primer. The *atu4544* coding region (666–1,173 bp) and its downstream 507 bp sequence were amplified from *A. tumefaciens* C58. The three fragments were ligated to the enzyme-digested suicide vector pEX18Km via homologous recombination. The correctly constructed pEX18Km-*atu4544-lacZ* chosen from *E. coli* DH5α was transformed into *A. tumefaciens* C58 and *A. tumefaciens* Δ*atu4545. A. tumefaciens atu4544-lacZ* and *A. tumefaciens* Δ*atu4545 atu4544-lacZ* generated by the gene replacement system were verified by PCR and DNA sequencing.

To evaluate the effects of *atu4545* on its promoter activity, *A. tumefaciens lacZ* and *A. tumefaciens*Δ*atu4545 lacZ* strains were constructed. Using diluted *A. tumefaciens* C58 as the template, the promoter sequences of *atu4545* were amplified using Pr-*atu4545*-F/R primers (see Table S1). The promoter-free pCB301 plasmid was digested with the restriction enzymes HindIII and BamHI. The purified promoter and *lacZ* reporter were ligated with the pCB301 plasmid and transferred into *E. coli* DH5α. The validated pCB301::promoter*_atu4545_::_lacZ_* plasmid was transformed into both *A. tumefaciens* C58 and *A. tumefaciens* Δ*atu4545*.

### Strain cultivation

To investigate *A. tumefaciens* growth on different carbon sources, a single colony of each *A. tumefaciens* strain retrieved from MG/L agar was incubated overnight in a tube of MG/L liquid medium at 28°C and 200 rpm. The bacteria grown to logarithmic phase were washed twice and adjusted to an OD_600_ of 0.5 using sterile water. One milliliter aliquots of each dilute bacterial suspension was transferred to 50 mL of AB minimal medium in a 250 mL shake flask containing 5 mM 4-hydroxybenzoate, 10 mM protocatechuate, 15 mM sucrose, or 15 mM L-arabinose as the sole carbon source. The absorbance value of OD_600_ was measured to monitor cell growth in aliquots of each culture collected every 4 h over time.

For protein expression, single colonies of *E. coli atu4544* and *E. coli atu4545* activated from LB solid plates were cultivated overnight at 37°C in LB tubes. Ampicillin (100 mg/L) or kanamycin (50 mg/L) was added as required. *E. coli atu4544* was inoculated into 150 mL LB medium (5% v/v inoculum), and *E. coli atu4545* was inoculated into 100 mL LB medium (2% vol/vol inoculum). Both were shaken at 200 rpm. Following the respective addition of 0.1 mL of 0.1 M IPTG and 0.7 mL of 0.1 M IPTG, when the OD_600_ reached 0.6, *E. coli Atu4544* and *E. coli Atu4545* were cultivated at 16°C for 16 h and 25°C for 4 h at 160 rpm.

To evaluate *A. tumefaciens* tumorigenicity, a single colony of *A. tumefaciens* strains (*A. tumefaciens* C58, Δ*atu4544*, Δ*atu4545*, C-Δ*atu4544*, and C-Δ*atu4545*) that grew on MG/L agar was retrieved and incubated overnight in a tube of MG/L liquid medium at 28°C and 200 rpm. Aliquots from each culture were transferred to AB-sucrose medium and grown to the logarithmic growth phase. The OD_600_ of each bacterial suspension was adjusted to 0.5 with sterile water for inoculation into the host plants.

To examine the growth of *A. tumefaciens* C58, Δ*atu4544*, Δ*atu4545*, C-Δ*atu4544*, and C-Δ*atu4545* under varying concentrations of 4-hydroxybenzoate, a single colony of each *A. tumefaciens* strain recovered from MG/L agar was cultured overnight in a tube of MG/L liquid medium at 28°C and 200 rpm. After two rounds of washing, the bacteria that had reached the logarithmic phase were adjusted to an OD_600_ of 0.5 using sterile water. 50 mL of AB minimum medium in a 250 mL shake flask with 0.5 g/L sucrose and 0, 0.02, 0.2, 2, 4, 6, 8, 10, 12, 14, or 16 mM of 4-hydroxybenzoate was filled with one milliliter aliquot of each diluted bacterial suspension. To track cell development in aliquots of each culture that was taken, the absorbance value of OD_600_ was recorded.

### Pot assays

The growth of *A. tumefaciens* C58, Δ*atu4544*, Δ*atu4545*, C-Δ*atu4544*, and C-Δ*atu4545* on 4HBA-containing plates was investigated. Cells recovered from overnight cultures in MG/L medium were washed twice with AB buffer and adjusted to an OD_600_ of 0.1. Each suspension was diluted by 10^−6^, and aliquots were spotted onto AB-sucrose agar plates containing 2, 4, 6, 8, or 10 mM of 4-hydroxybenzoate. The growth phenotype of the *A. tumefaciens* strains on the 4HBA-containing agar was observed after 48 h of cultivation at 28°C.

### Protein expression and purification

The *atu4544* and *atu4545* genes were amplified from diluted *A. tumefaciens* C58. The *atu4544* gene was cloned into the pGEX-4T-1 expression vector with an N-terminal GST-tag. The *atu4545* gene was cloned into the pET30a vector with an N-terminal 6-histidine tag. The pET30a::HIS-*atu4545* and pGEX::GST-*atu4544* were, respectively, transformed in *E. coli* BL21 (DE3) to generate *E. coli atu4544* and *E. coli atu4545*. After recombinant protein expression in the two strains was induced by IPTG, the cells were collected, washed, and resuspended in phosphate-buffered saline (PBS; 10 mM, pH 7.4). After lysis by sonication in cycles of 4 s ON and 8 s OFF for 40 min, each cell suspension was centrifuged (12,000 × *g*, 4°C for 20 min). Each collected supernatant was passed through a 0.45 µm microfiltration membrane for protein purification. The supernatants of *E. coli atu4544* and *E. coli atu4545* were, respectively, incubated with BeyoGold GST-tag Purification Resin and BeyoGold His-tag Purification Resin for 1 h at 4°C with gentle rocking. The resins were washed several times with washing solution (His-tag resin: 1 L containing Tris-base 6.06 g, NaCl 29.22 g, pH 8.0; GST-tag resin: 1 L containing NaCl 8.19 g, KCl 201 mg, Na_2_HPO_4_ 1.42 g, KH_2_PO_4_ 245 mg, pH 8.0) to remove unbound and nonspecifically bound proteins. GST-atu4544 and HIS-atu4545 were eluted using 250 mM imidazole and 10 mM GST, respectively. Protein samples were analyzed using 12% sodium dodecyl sulfate-polyacrylamide gel electrophoresis.

### Electrophoretic mobility shift assays

The interaction of purified HIS-atu4545 with the intergenic sequences of *atu4544* and *atu4545* was evaluated by EMSA. Unlabeled intergenic sequences and unrelated DNA (similar length) were, respectively, amplified from diluted *A. tumefaciens* C58 using int-4544-F/R and *pobR*-inter-F/R primers. The recovered PCR products provided templates for amplifying the FAM [5(6)-carboxyfluorescein]-labeled intergenic sequence and unrelated sequences with the primer pairs int-4445-F/FAM and *pobR*-inter-F/FAM. The sites for atu4545 protein binding to the *atu4544* promoter were predicted using the Motif Alignment and Search Tool from a set of motifs for PobR binding in other species (20, 22, 23, 27). A FAM-labeled intergenic sequence containing mutant binding sites was synthesized (Table S1). Unrelated DNA and bovine serum albumin were added to some lanes for the binding assay as negative controls. The 50 mL of binding buffer contained 0.394 g Tris-base, 0.186 g KCl, 0.019 g EDTA•2Na, 10% glycerol, and 0.008 g dithiothreitol, pH 8.0. The 20 µL reaction system that was added contained 20 ng DNA probes, 0.1 µg salmon sperm DNA, an increasing amount of HIS-atu4545 protein (0, 1, 4, and 6 µM), and a binding buffer. Each reaction volume was then incubated at 25°C for 40 min. Pre-electrophoresis using 6% native PAGE gels was performed at 20 V for 30 min, followed by protein separation at 100 V for 1.5 h. Each gel was scanned using a Typhoon FLA 9500 device (GE Healthcare, Uppsala, Sweden).

### Enzymatic activity assay

4-Hydroxybenzoate hydroxylase activity was determined as previously described (47). The reaction products and substrates were detected by HPLC. The 3 mL reaction mixture in 50 mM Tris∙Cl (pH 7.4) buffer contained 5 µM purified GST-atu4544 protein, 0.5 mM 4-hydroxybenzoate, 0.4 mM NADPH (or 0.4 mM NADH), and 5 µM FAD. Each mixture was incubated at 30°C. In the reaction, 4-hydroxybenzoate consumption and protocatechuate formation were catalyzed by 4-hydroxybenzoate hydroxylase encoded by the atu4544 protein. The 500 µL reaction mixtures collected at 0, 0.5, and 1 h were put in 100°C boiling water for 5 min to quench the reaction. After removing precipitated protein using Ultra 30K cutoff centrifugal filters (Millipore, Billerica, MA, USA), 20 µL aliquots of each reaction mixture were measured by HPLC at 250 nm using a ZORBAX SB 80 Å C18 column, eluted with acetonitrile-water (9:91, vol/vol, with the pH adjusted to 2.5 using phosphoric acid) at a flow rate of 1 mL min^−1^. One unit of 4-hydroxybenzoate hydroxylase-specific activity was defined as the amount of enzyme that generated 1 µmol min^−1^ protocatechuate per mg protein at 30°C.

β-galactosidase activity was determined as previously described (48) with some modifications. Briefly, a single colony of each *A. tumefaciens* strain on MG/L agar was incubated overnight in a tube of MG/L liquid medium at 28°C and 200 rpm. After washing twice using sterile water, two procedures were performed depending on the sample. In the first procedure, 100 µL of the suspension of *A. tumefaciens atu4544-lacZ*, *A. tumefaciens* Δ*atu4545 atu4544-lacZ*, *A. tumefaciens lacZ*, and *A. tumefaciens* Δ*atu4545 lacZ* was inoculated in 5 mL AB-arabinose medium, followed by the addition of 10 mM protocatechuate, 5 mM 4-hydroxybenzoate, or 15 mM adipic acid as needed. The OD_600_ values of each bacterial suspension were measured when the cells reached the logarithmic growth stage. In the second procedure, 100 µL of the suspension of *A. tumefaciens virB-lacZ Δatu4544* and *A. tumefaciens virB-lacZ Δatu4545* was inoculated in 5 mL AB-sucrose medium or 5 mL AB-sucrose medium containing 5 mM 4-hydroxybenzoate. After 4 h of cultivation, 100 µM acetosyringone was added. After continuous cultivation for 9 h, the OD_600_ of each bacterial suspension was measured. Five hundred microliters of each bacterial suspension was successively received 500 µL Z-buffer, 50 µL chloroform, and 20 µL 0.1% SDS. The reaction was started by adding 200 µL of o-nitrophenyl-β-galactoside and was terminated after the solution turned yellow by adding 1 M Na_2_CO_3_. Reaction time is the time it takes for a batch of experimental samples to turn yellow from the start of the reaction. After centrifugation, the OD_420_ of each supernatant was measured. One unit of β-galactosidase activity was defined as the amount of enzyme required to degrade o-nitrophenyl-β-galactoside to produce 1 µmol min^−1^ o-nitrophenol at 37°C.

### Tumorigenesis assay

A tumorigenesis assay was performed to determine tumor formation and bacterial abundance in tumors using *A. tumefaciens* infections of carrot plates and kalanchoe leaves (49, 50). The carrots were cleaned and their middle parts were peeled. Cylindrical disks with an inner diameter of 1.5 cm and a thickness of 5 mm were made from the clean and peeled middle parts of the carrots. After soaking in 1.05% sodium hypochlorite for 30 min, the disks were placed on 1.5% agar solid plates at regular intervals. Five microliter aliquots of each bacterial suspension was inoculated in the center of each carrot disk. Fifteen replicates were analyzed for each bacterial strain. The infected carrot tuber discs were incubated at 25°C for 2–4 weeks. Kalanchoe leaves were wiped using a 75% alcohol swab and scratched with a hypodermic needle to create wounds of similar length and depth. Five microliter aliquots of each bacterial suspension was inoculated on each wound line of kalanchoe leaves at room temperature in a cool and shaded area. *A. tumefaciens* C58, NT1, Δ*atu4544*, Δ*atu4545*, C-Δ*atu4544*, and C-Δ*atu4545* were individually injected on the same leaf to rule out the possibility of leaf age-related tumorigenesis. The tumors were carefully scraped off the discs or incisions after 2–4 weeks and weighed. In addition, 0.1 g of tumors from carrots or kalanchoe leaf was put in 500 µL sterile normal saline and ground for as close to the same duration and with the same force as possible. One hundred microliter aliquots of each mixture was diluted by 10^−8^, and 100 µL of each bacterial suspension was spread onto AB-sucrose agar. The colonies on the plates were counted after 48 h at 28°C. To determine 4-hydroxybenzoate concentrations in tumors, on each carrot disk, 50 µL of 15 mM 4-hydroxybenzoate was injected into the tumors once they had grown out (after approximately 10 days). These disks were incubated for 4 weeks. The tumors were collected, weighed, colonies were enumerated, and 4-hydroxybenzoate concentrations were determined by HPLC.

## SUPPLEMENTAL MATERIAL

Supplemental material aem.00255-25-s0001.docx Table S1; Figures S1 to S10.

ASM does not own the copyrights to Supplemental Material that may be linked to, or accessed through, an article. The authors have granted ASM a non-exclusive, world-wide license to publish the Supplemental Material files. Please contact the corresponding author directly for reuse.
