# Supplementary material for: Function and regulation of pob genes for 4-hydroxybenzoate catabolism in Agrobacterium tumefaciens
Source: Appl Environ Microbiol. 2025 Jun 3;91(7):e00255-25. doi: 10.1128/aem.00255-25 (PMC12285266; doi:10.1128/aem.00255-25)
Supplement: Supplemental material — Table S1; Figures S1 to S10. [file aem.00255-25-s0001.docx]

***Supplementary Materials***

**Function and Regulation of *pob* Genes for 4-hydroxybenzoate Catabolism in *Agrobacterium tumefaciens***

Nan Xu^1^, Wanyu Wang^1^, Shuang Cheng^1^, Jiaojiao Zuo^1^, Minliang Guo^1^*

^1^College of Bioscience and Biotechnology Yangzhou University, Yangzhou 225009, China

* **Correspondence:**

Corresponding Author

guoml@yzu.edu.cn

**Table S1 The primers used in this study**

| Primer name | Sequence（5^，^-3^，^） | Application |
| --- | --- | --- |
| *atu4544*-U-F | GCCAGTGCCAAGCTTGAAAAGGTGCGCTTCGTG | To amplify the upstream 500 bp sequence of *atu4544* gene |
| *atu4544*-U-R | GACAAGTTCGGGAGGACATCGCGTTTTGAGGAAAAGTGTGAAG |  |
| *atu4544-*D-F | CTTCACACTTTTCCTCAAAACGCGATGTCCTCCCGAACTTGTC | To amplify the downstream 500 bp sequence of *atu4544* gene |
| *atu4544-*D-R | GGTACCCGGGGATCCTCTCGGCAAAATCCCGTAAAC |  |
| *atu4545*-U-F | GCCAGTGCCAAGCTTACATTGCCGGCGTCATAG | To amplify the upstream 500 bp sequence of *atu4545* gene |
| *atu4545*-U-R | TGGCGAGAATGCTGGTCTGACTCACCATCACTCCTCCGT |  |
| *atu4545-*D-F | CACGGAGGAGTGATGGTGAGTCAGACCAGCATTCTCGC | To amplify the upstream 631 bp sequence of *atu4545* gene |
| *atu4545-*D-F | GGTACCCGGGGATCCCCTATGACGGCAAGTTCTTC |  |
| C*-atu4544*-F | GATTACGCCAAGCTTGATGCGTACACAGGTCGTC | To amplify the sequence of *atu4544* inserting to pUCA19 |
| C*-atu4544*-R | GGTACCCGGGGATCCTCAATAAGGAAGCCCCACAT |  |
| C*-atu4545*-F | GATTACGCCAAGCTTGATGCGGGTGACTGCCGATG | To amplify the sequence of *atu4545* inserting to pUCA19 |
| C*-atu4545*-R | GGTACCCGGGGATCCTCAGCGGGAACTGGCGAGA |  |
| Pr-*atu4545*-F | AGAACTAGTGGATCCCATGATGTCCTCCCGAACTTGT | To amplify the 121bp promoter of *atu4545* gene inserting to pCB301 |
| Pr-*atu4545*-R | GTGAATCCGTAATCATGGTCATCTCACCATCACTCCTCCGTG |  |
| Pr-*atu4545-lacZ*-F | CACGGAGGAGTGATGGTGAGATGACCATGATTACGGATTCAC | To amplify the lacZ encoding sequence and link it to the downstream of the above 121 bp promoter |
| Pr-*atu4545-lacZ* -R | CTTGATATCGAATTCTTATTTTTGACACCAGACCAACT |  |
| pGEX-atu4544-F | GTTCCGCGTGGATCCATGCGTACACAGGTCGT | To amplify the sequence of *atu4544* inserting to pGEX-4T-1 |
| pGEX-atu4544-R | TCGAGTCGACCCGGGTCAATAAGGAAGCCCCACAT |  |
| pET30a-4545-F | GCTGATATCGGATCCATGCGGGTGACTGCCGATG | To amplify the sequence of *atu4545* inserting to pET30 |
| pET30a-4545-R | TGCGGCCGCAAGCTTTCAGCGGGAACTGGCGAGA |  |
| FAM | FAM-TGCCTGCAGGTCGACGAT | The FAM label for EMSA |
| int-4445-F | CATCTCACCATCACTCCTCC | To amplify the upstream 118 bp sequence of atu4544 with FAM label. |
| int-4445-R | TGCCTGCAGGTCGACGATGATGTCCTCCCGAACTTGTC |  |
| pobR-inter-F | CATCGTGACGATCACTCCAC | To amplify 118 bp unrelated sequence with the bind of atu4545 |
| pobR-inter-R | TGCCTGCAGGTCGACGATCGATGCGTTGCTGGAAG |  |
| int-tb-118 | TGCCTGCAGGTCGACGATCATCTCACCATCACTCCTC**GCTGGAAGGCCGCATCG**GTGCAATTTACAGACAGAAAAGTCCATTGAGCTTGTGGCCGGTCTTGGTCAATTTCTTGACCGGAGACAAGTTCGGGAGGAC | Synthetic DNA fragments; The binding sequence was replaced by other 16 bp sequence in bold. |
| atu4544-508-F | ACGACGGCCAGTGCCAAGCTTCATTCAATGTTCGCTGGATGAC | To amplify the upstream 508 bp of *atu4544* gene |
| atu4544-508-R | CGTAATCATGGTCATTTTCTCCTCTTTTCAATAAGGAAGCCCCACATAAT |  |
| *atu4544*-*LacZ*-F | GGGGCTTCCTTATTGAAAAGAGGAGAAAATGACCATGATTACG | lacZ gene coding frame was amplified |
| *atu4544*-*LacZ*-R | GCGCACCTTTTCCGAATAGTTATTTTTGACACCAGACCAACTGG |  |
| atu4544-D507-F | CCAGTTGGTCTGGTGTCAAAAATAACTATTCGGAAAAGGTGCGCTT | 507bp downstream of *atu4543* gene was amplified |
| atu4544-D507-R | GAGAGCGGTACCCGGGGATCCGCGTTTTGAGGAAAAGTGTGAAG |  |

The sequence of the intergenic region of atu4544 and atu4545 used as a DNA probe: CATCTCACCATCACTCCTCCGTGCGATGGTCGGATTGTGCAATTTACAGACAGAAAAGTCCATTGAGCTTGTGGCCGGTCTTGGTCAATTTCTTGACCGGAGACAAGTTCGGGAGGACATCATCGTCGACCTGCAGGCA-FAM


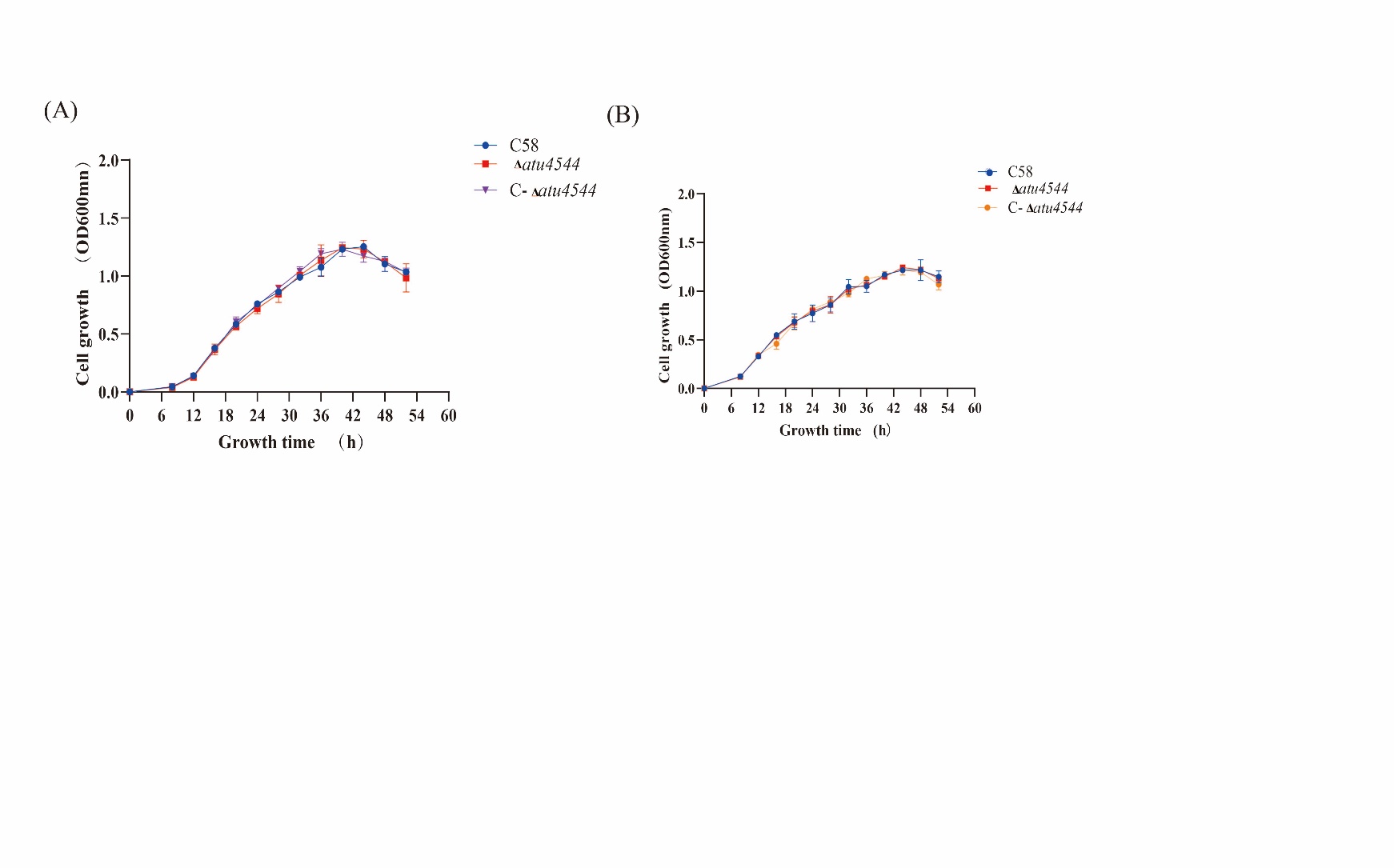


**Figure S1. Cell growth of *A. tumefaciens* strains (C58, Δ*atu4544*, C-Δ*atu4544*) on common *AB* minimal medium .** (A) On 15 mM sucrose (B) On 15 mM arabinose

**
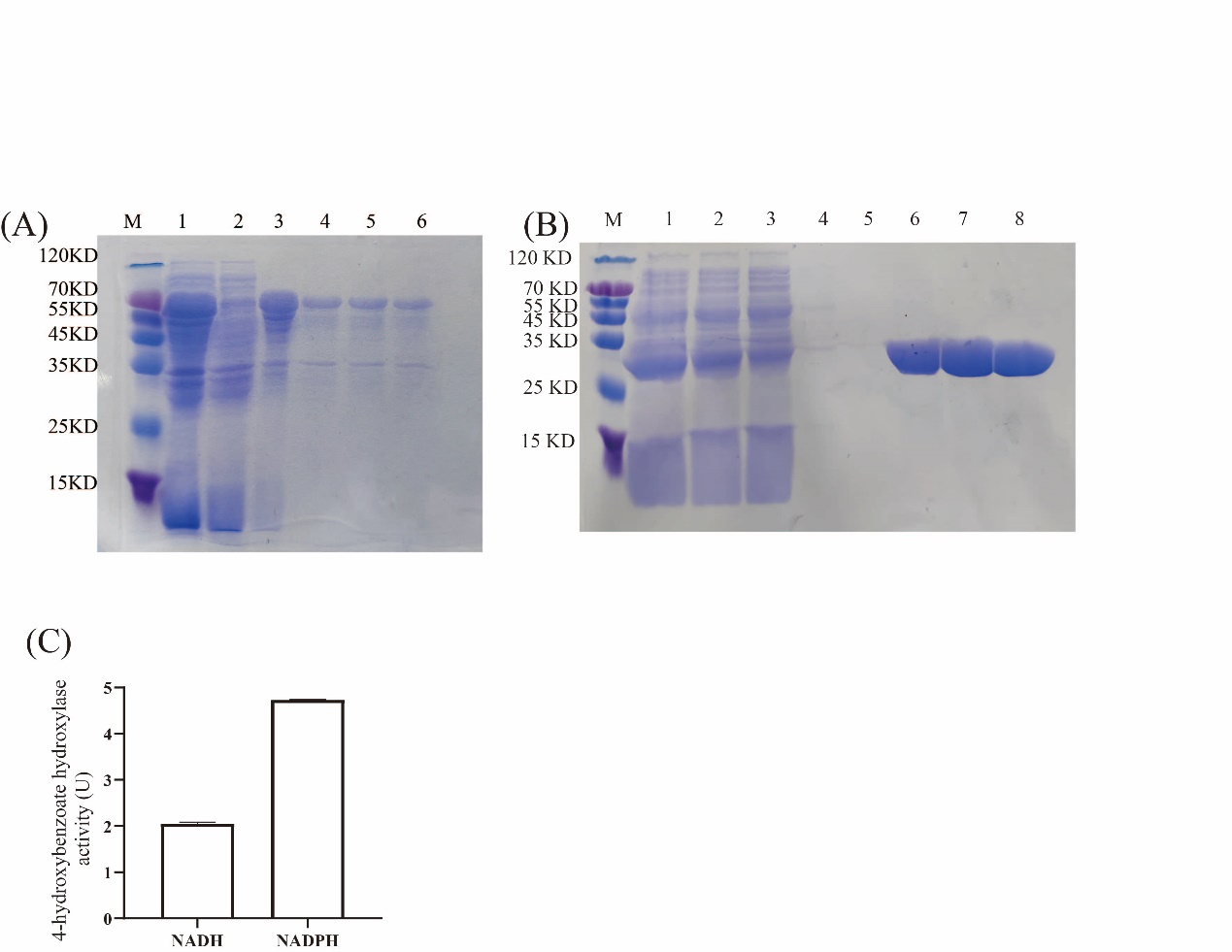
**

**Figure S2. Protein expression and purification of atu4544-GST protein**

(A) Protein expression and purification of atu4544-GST protein. M: protein marker, lane 1: lysed cells, lane 2: supernatant of lysed cell preparation, lane 3: precipitate of lysed cell preparation, lane 4-6: target protein eluent. (B) Protein expression and purification using empty plasmid pGEX-4T1. M: protein marker, lane 1: lysed cells, lane 2: supernatant of lysed cell preparation, lane 3: precipitate of lysed cell preparation, lane 4-5: lysate draining fluid, lane 6-8: target protein eluent.


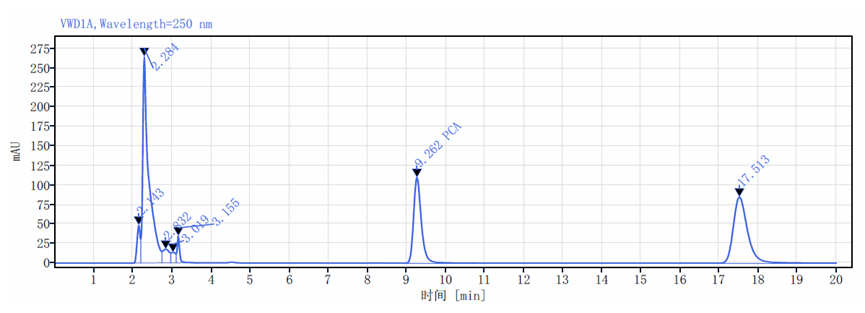

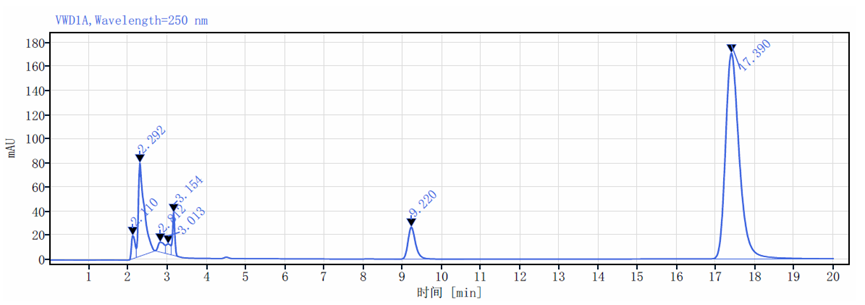

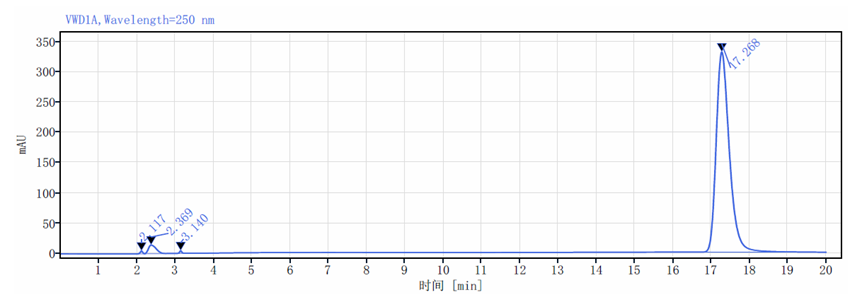
**
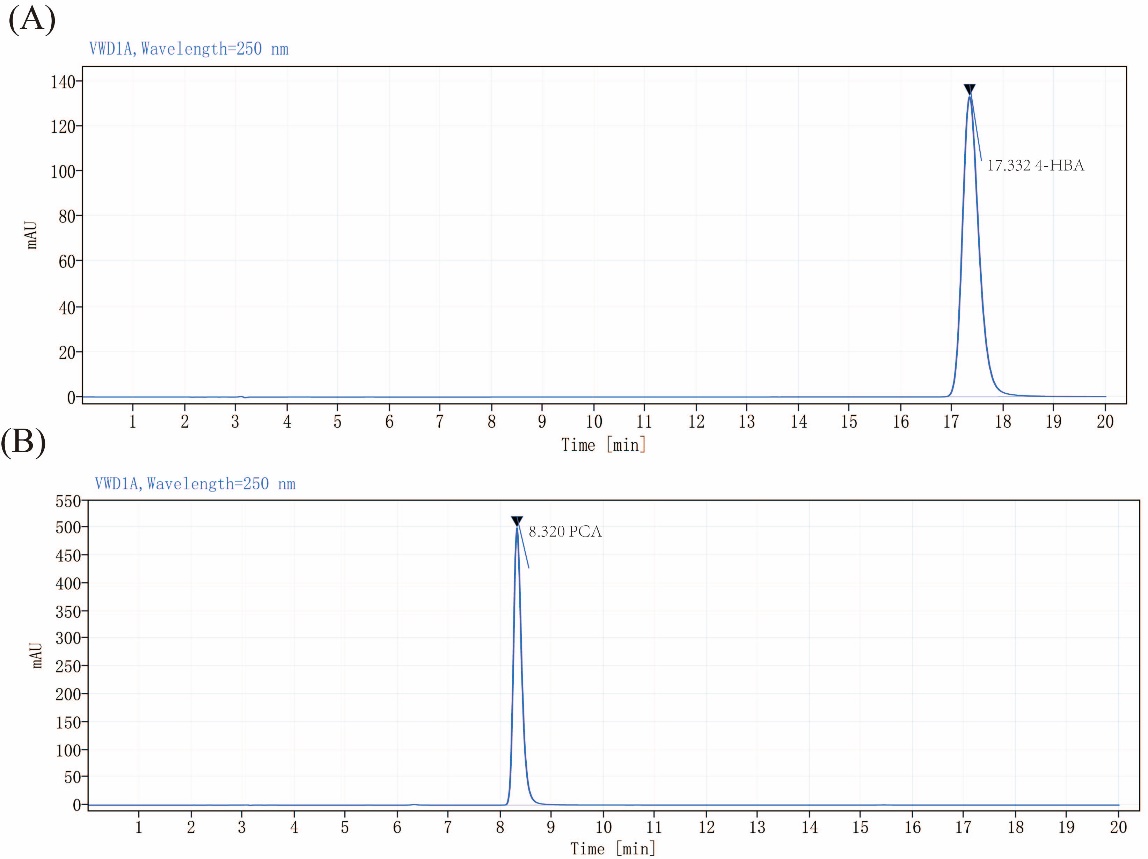
**

(E)

(D)

(C)

**Figure S3. HPLC chromatogram of the reaction’s product and substrate catalyzed by pobA (atu4544)** (A) HPLC chromatogram of 4-hydroxybenzoate. (B) HPLC chromatogram of protocatechuate. (C) HPLC chromatogram of reaction mixtures adding purified GST-tag. (D) HPLC chromatogram of reaction mixtures adding purified atu4544 protein at 0 h. (E) HPLC chromatogram of reaction mixtures adding purified atu4544 protein at 0.5 h.

**
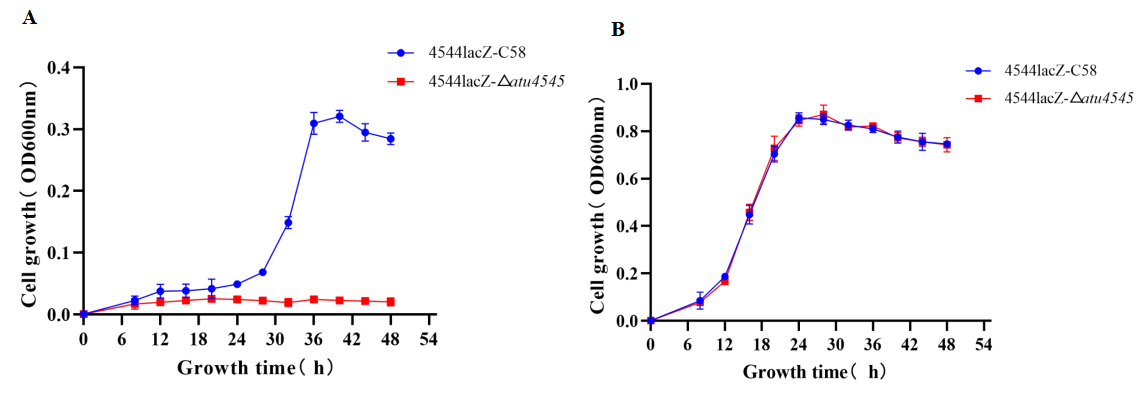
**

**Figure S4. Cell growth of LacZ *in situ* insertion of *Agrobacterium tumefaciens* strains.** (A) On 5mM 4-hydroxybenzoate (B) 10mM protocatechuic acid. 4544lacZ-C58 and 4544lacZ-Δ*atu4545* respectively correspond to *A. tumefaciens lacZ* and *A. tumefaciens* Δ*atu4545* *lacZ*. Every point represents a standard deviation from the mean of a minimum of three replicates.

M 1 2 3 4 5 6 7 8

**
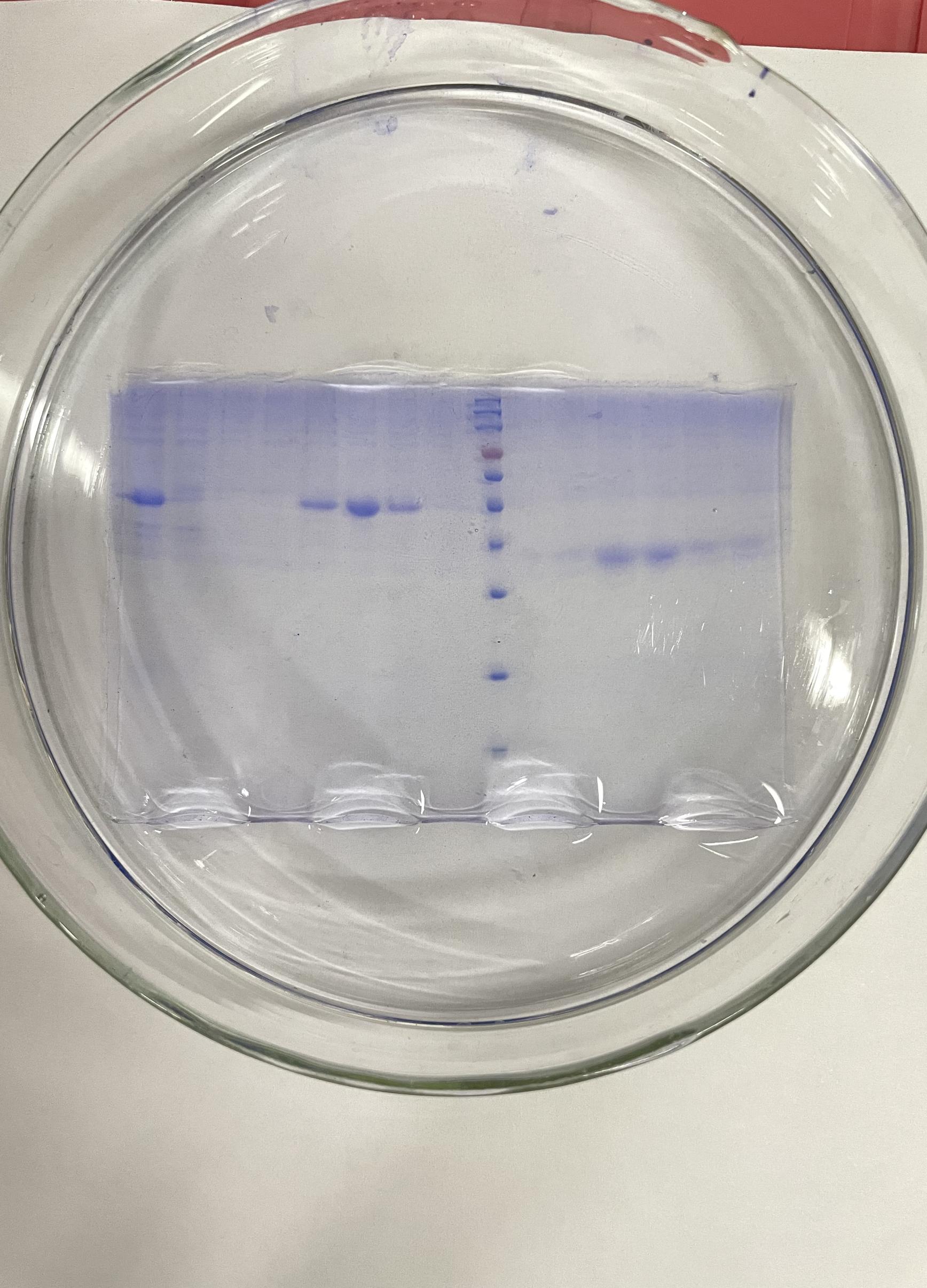
**

130KDa

180KDa

100KDa

72KDa

55KDa

43KDa

33KDa

25KDa

17KDa

8KDa

**Figure S5 Protein expression and purification of atu4545-HIS protein**

M：Protein Marker, lane1: eluent using 500 mM imidazole, lane 2-4: target protein eluent, 5: wash buffer, lane 6: flow-through using non-denaturing lysis buffer, lane 7：flow-through，lane 8：supernatant of lysed cell preparation


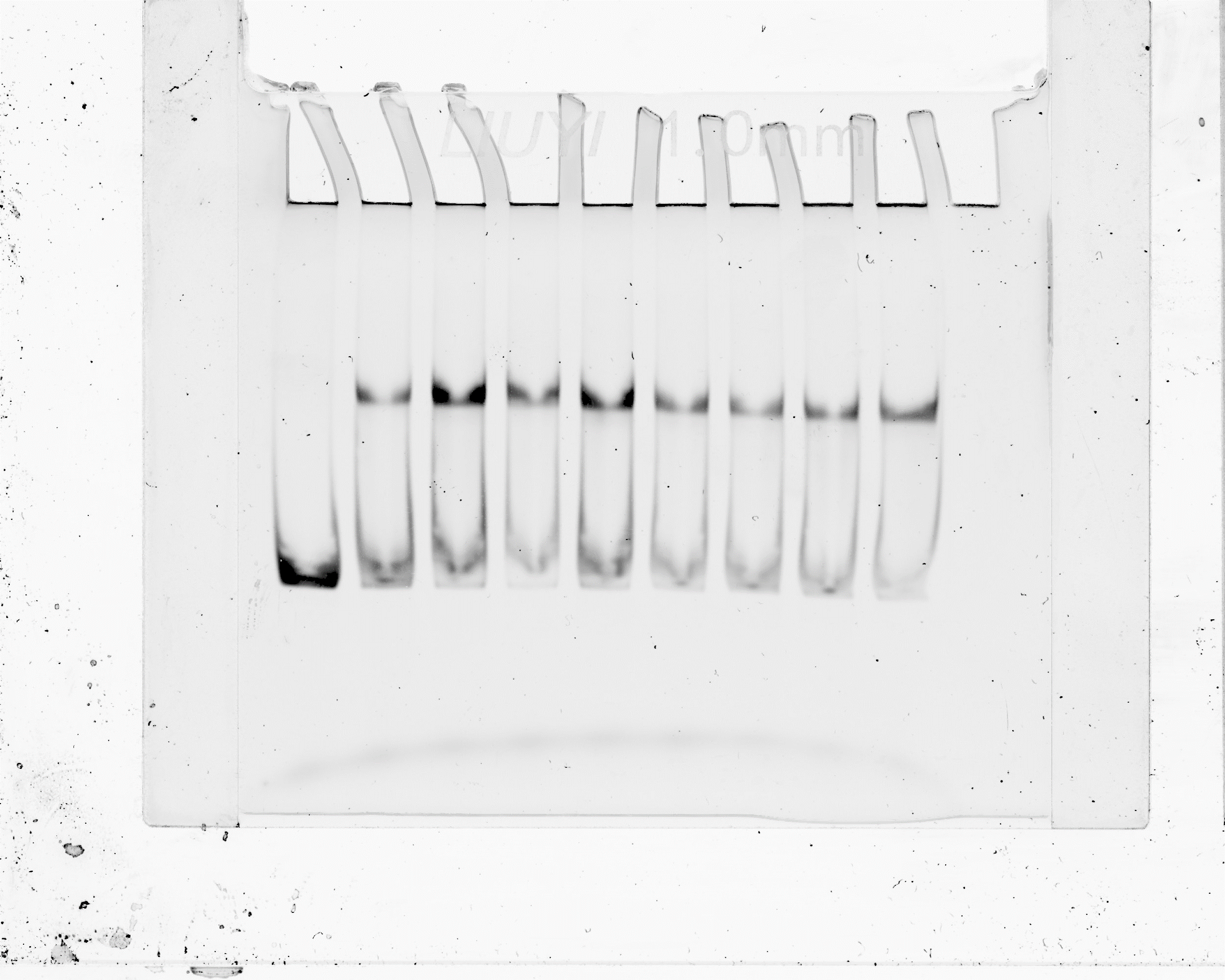


(A)

4HBA(mM) 0 0 0.25 0.5 1.0 2.0

PobR-his(4 𝜇M) - + + + + +

DNA (20 ng) + + + + + +

(B)

AA (Mm) 0 0 0.25 0.75 1.5 2.5 5.0

PobR-his(4 𝜇M) - + + + + + +

DNA (20 ng) + + + + + + +


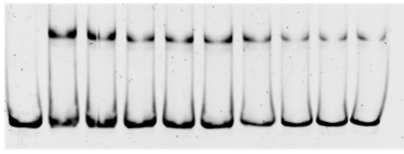


**Figure S6. EMSA results of pobR with the FAM-labeled DNA at different concentrations of effectors** (A) The effects of 4-hydroxybenzoate (4HBA) (B) The effects of adipic acid (AA) The FAM-labeled DNA probe is the intergenic region of atu4544 and atu4545.


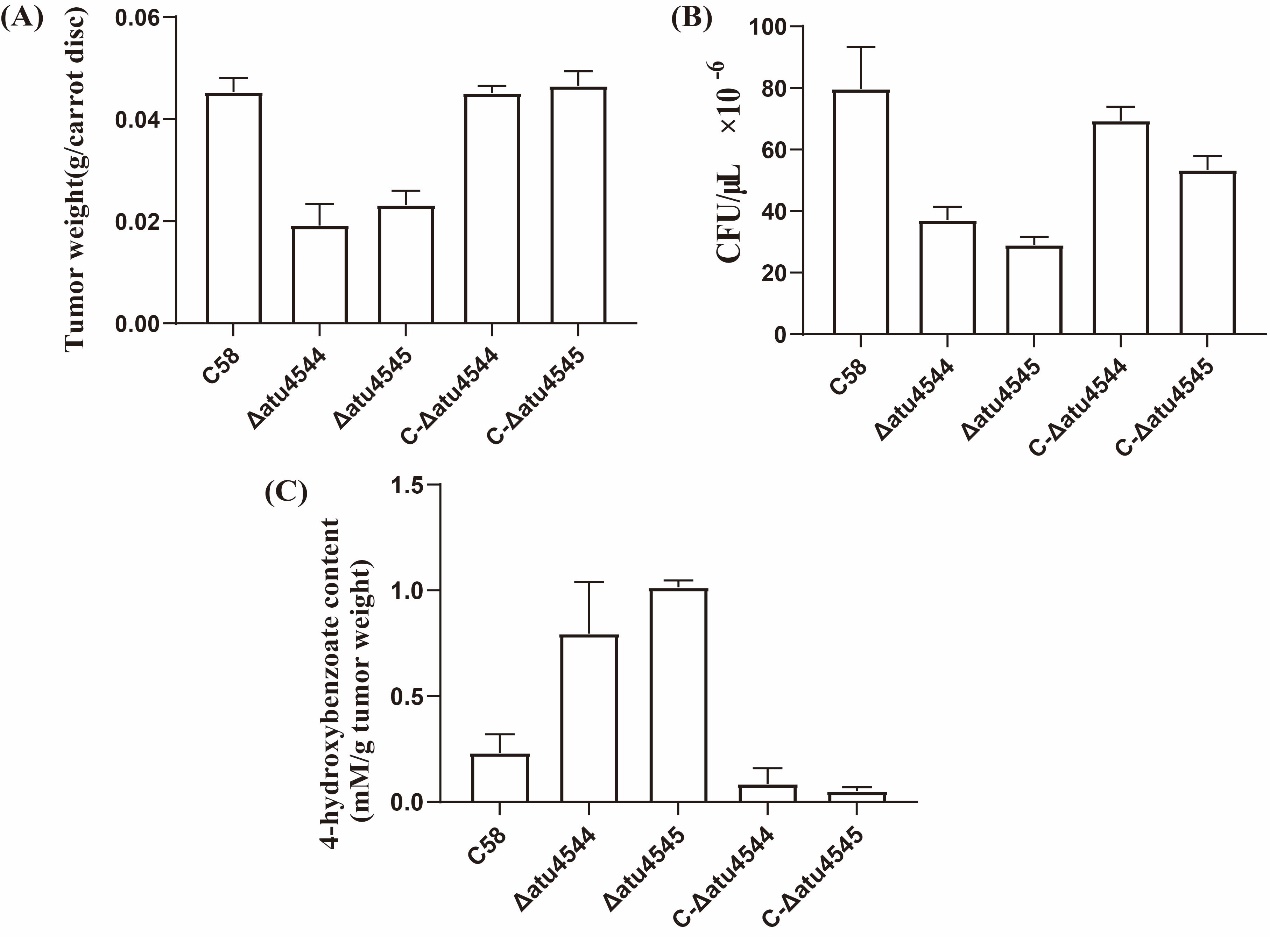


**Figure S7. Effects of atu4544 and atu4545 on tumorigenesis on the carrots discs when exogenous added 4-hydroxybenzoate** (A) Tumor weights after infecting the carrots for 4 weeks (B) The number of colonies in tumors on carrots infected by *A. tumefaciens* strains after 4 weeks (C) 4-hydroxybenzoate contents in the tumors after 4 weeks.

**
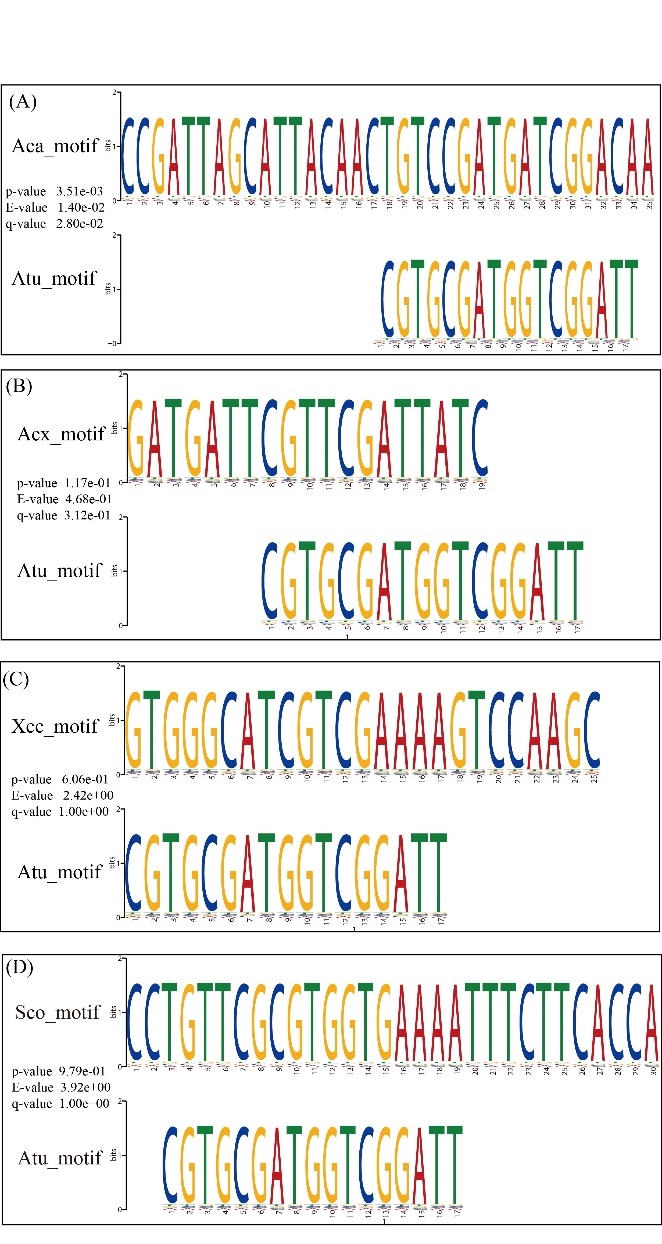
**

**Figure S8. The comparison between the *atu4545* binding sequences with other known regulatory motifs of *pobR*.**

Aca, Atu, Acx, Xcc, and Sco respectively represent *Acinetobacter calcoaceticus*, *A. tumefaciens*, *Azotobacter chroococcum*, *Xanthomonas campestris*, and *Streptomyces coelicolor*.

**
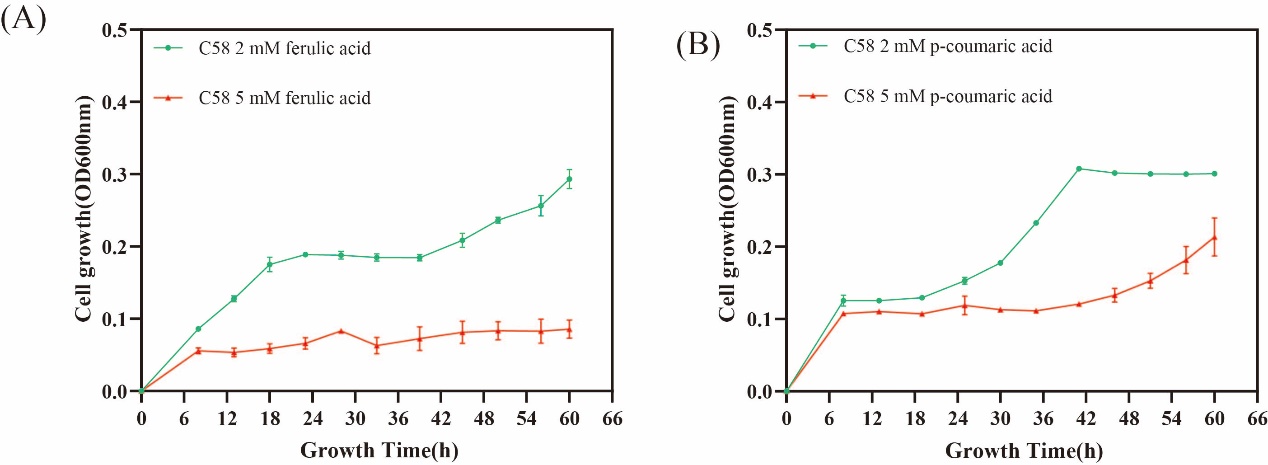
**

**Figure S9. Cell growth of *A. tumefaciens* C58 on AB minimal medium using aromatic compounds as sole carbon sources** (A) Effects of different concentrations of ferulic acid (B) Effects of different concentrations of *p*-coumaric acid

**
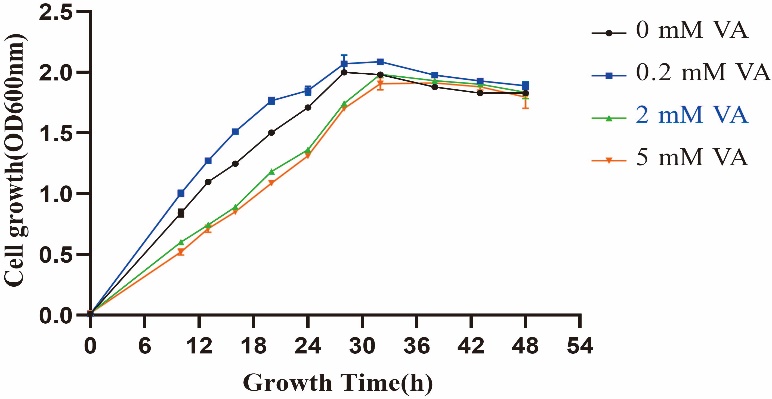
**

**Figure S10. Growth curves of *A. tumefaciens* C58 on AB-sucrose medium adding different concentrations of Vanillic acid (VA)**
